# MicroRNA-146a-mediated downregulation of IRAK1 protects mouse and human small intestine against ischemia/reperfusion injury

**DOI:** 10.1002/emmm.201201298

**Published:** 2012-11-09

**Authors:** Cécilia Chassin, Cordelia Hempel, Silvia Stockinger, Aline Dupont, Joachim F Kübler, Jochen Wedemeyer, Alain Vandewalle, Mathias W Hornef

**Affiliations:** 1 Institute of Medical Microbiology and Hospital Epidemiology, Hannover Medical SchoolHannover, Germany; 2 Department of Pediatric Surgery, Hannover Medical SchoolHannover, Germany; 3 Department of Gastroenterology and Hepatology, Hannover Medical SchoolHannover, Germany; 4 INSERM U773, Centre de Recherche Biomédicale Bichat-Beaujon (CRB3), Université Paris 7-Denis DiderotParis, France

**Keywords:** inflammation, intestine, Irak1, ischemia–reperfusion, microRNA

## Abstract

Intestinal ischemia/reperfusion (I/R) injury causes inflammation and tissue damage and is associated with high morbidity and mortality. Uncontrolled activation of the innate immune system through toll-like receptors (Tlr) plays a key role in I/R-mediated tissue damage but the underlying mechanisms have not been fully resolved. Here, we identify post-transcriptional upregulation of the essential Tlr signalling molecule interleukin 1 receptor-associated kinase (Irak) 1 as the causative mechanism for post-ischemic immune hyper-responsiveness of intestinal epithelial cells. Increased Irak1 protein levels enhanced epithelial ligand responsiveness, chemokine secretion, apoptosis and mucosal barrier disruption in an experimental intestinal I/R model using wild-type, *Irak1*^−/−^ and *Tlr4*^−/−^ mice and ischemic human intestinal tissue. Irak1 accumulation under hypoxic conditions was associated with reduced K48 ubiquitination and enhanced Senp1-mediated deSUMOylation of Irak1. Importantly, administration of microRNA (miR)-146a or induction of miR-146a by the phytochemical diindolylmethane controlled Irak1 upregulation and prevented immune hyper-responsiveness in mouse and human tissue. These findings indicate that Irak1 accumulation triggers I/R-induced epithelial immune hyper-responsiveness and suggest that the induction of miR-146a offers a promising strategy to prevent I/R tissue injury.

## INTRODUCTION

Ischemia/reperfusion (I/R) injury is observed in a variety of diseases, such as vascular occlusion, haemorrhagic shock or trauma. It is also an unavoidable event during organ transplantation. Reperfusion of post-ischemic tissue induces activation of the innate immune system, leading to an inflammatory response that significantly contributes to the hypoxic cell damage (Chen et al, 2003; Mkaddem et al, 2010; Watson et al, 2008). An important role of Toll-like receptor (Tlr) activation in the pathogenesis of I/R injury has been established in post-ischemic hepatic, cardiac, renal tissue damage and haemorrhagic shock (Bamboat et al, 2010; Ellett et al, 2009; Moses et al, 2009; Suzuki et al, 2008; Zanotti et al, 2009). Particularly, a critical role of the Tlr4 signalling pathway has been identified (Moses et al, 2009; Pope et al, 2010; Watson et al, 2008). However, the underlying molecular mechanism of the exaggerated innate immune response following transient ischemia has remained poorly understood.

Innate immune signalling at the intestinal epithelium actively contributes to antimicrobial host defence and the maintenance of mucosal homeostasis (Cario et al, 2007; Nenci et al, 2007; Rakoff-Nahoum et al, 2004; Voss et al, 2006; Weiss et al, 2004; Zaph et al, 2007). Given the permanent exposure to the enteric microbiota, efficient control of epithelial innate immune activation is, however, required to prevent inappropriate cell stimulation, tissue inflammation and organ dysfunction (Chassin et al, 2010; Turer et al, 2008; Vereecke et al, 2010; Xiao et al, 2007). We recently demonstrated that downregulation of the essential Tlr signalling molecule interleukin 1 receptor-associated kinase 1 (Irak1) in intestinal epithelial cells (IEC) contributes significantly to protect the immature intestinal epithelium from bacteria-induced tissue damage during the neonatal period (Lotz et al, 2006). Signalling-induced proteasomal degradation and translational repression as a result of enhanced microRNA (miR)-146a expression act in concert to reduce Irak1 and protect the intestinal mucosa against inappropriate stimulation during postnatal colonization (Chassin et al, 2010).

The observed tight regulation of Irak1 protein levels prompted us to investigate whether enhanced epithelial Irak1 protein might also occur and contribute to innate immune-mediated tissue damage in relevant clinical conditions. Given that innate immune hyper-responsiveness is found under clinical conditions of I/R, we hypothesized that the accumulation of Irak1 under ischemic conditions might contribute to the enhanced innate immune response after intestinal I/R. Our results identify a direct functional link between oxygen restriction, Irak1 protein accumulation and innate immune-mediated cell damage in mouse and human tissue *in vitro* and *in vivo* and characterize the underlying molecular mechanisms. Additionally, we provide *in vivo* evidence that the administration of miR-146a or the pharmacological induction of miR-146a prevent post-ischemic Irak1 upregulation and reduce innate immune hyper-responsiveness and I/R injury.

## RESULTS

### Hypoxia increases epithelial IRAK1 protein and innate immune responsiveness

**Figure 1:**
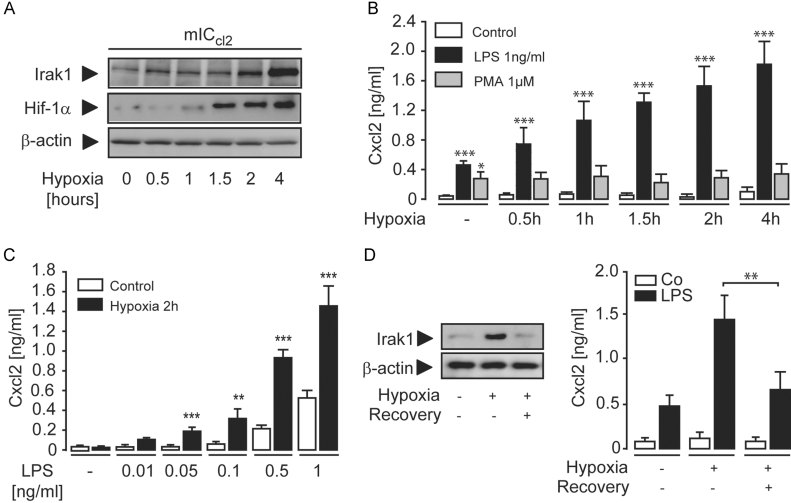
Hypoxia increases epithelial Irak1 protein and causes innate immune hyper-responsiveness *Student's *t*-test *p* < 0.05, **p < 0.01, ***p < 0.001 compared to controls (**B**,**C**) or hypoxia LPS-treated (**D**). Values are means ± SEM from 3 to 5 independent experiments, *n* = 4/group. Time kinetic of Irak1 and Hif-1α protein expression in mIC_cl2_ cells after incubation in hypoxic chambers.mIC_cl2_ cells were incubated in hypoxic chambers for indicated time, and subsequently stimulated under normoxic conditions with 1 ng/ml LPS or 1 µM PMA for 6 h, and the secretion of Cxcl2 was determined. For each data point, *n* = 4. normoxia: LPS 0.536 ± 0.048 and PMA 0.239 ± 0.091 *versus* control 0.063 ± 0.008, *p* = 2 × 10^−6^ and *p* = 0.09, respectively; 0.5 h: LPS 0.728 ± 0.117 *versus* control 0.073 ± 0.033, *p* = 3 × 10^−3^; 1 h: LPS 1.156 ± 0.325 *versus* control 0.094 ± 0.011, *p* = 6 × 10^−4^; 1.5 h: LPS 1.336 ± 0.144 *versus* control 0.065 ± 0.015, *p* = 2 × 10^−6^; 2 h: LPS 1.580 ± 0.291 *versus* control 0.037 ± 0.034, *p* = 4 × 10^−5^; 4 h: LPS 1.821 ± 0.425 *versus* control 0.117 ± 0.082, *p* = 2 × 10^−4^).mIC_cl2_ cells were incubated in hypoxic chambers for 2 h and subsequently stimulated with various concentrations of LPS under normoxic conditions for 6 h, and the secretion of Cxcl2 was determined. For each data point, *n* = 4.0.05: hypoxia 0.188 ± 0.040 *versus* control 0.032 ± 0.020, *p* = 4 × 10^−4^; 0.1: hypoxia 0.316 ± 0.098 *versus* control 0.059 ± 0.025, *p* = 0.002; 0.5: hypoxia 0.931 ± 0.080 *versus* control 0.215 ± 0.034, *p* = 3 × 10^−6^; 1: hypoxia 1.455 ± 0.201 *versus* control 0.525 ± 0.075, *p* = 10^−4^.mIC_cl2_ cells were left untreated or incubated in hypoxic chambers for 2 h followed for one fraction of cells by overnight incubation in fresh medium in normoxic conditions (recovery). Subsequently, the levels of Irak1 and Cxcl2 secretion were determined after 6 h stimulation with 1 ng/ml LPS. For each data point, *n* = 4. Hypoxia/recovery + LPS (0.690 ± 0.173) *versus* Hypoxia + LPS (1.456 ± 0.317), *p* = 0.005.

The response of intestinal epithelial m-IC_cl2_ cells to lipopolysaccharide (LPS) critically depends on the innate immune receptor Tlr4 and the signal molecule Irak1 (Chassin et al, 2010; Hornef et al, 2002). Consequently, siRNA-mediated downregulation of Irak1 or Tlr4 almost completely abolished LPS-induced NF-κB reporter activity and the secretion of the proinflammatory chemokine Cxcl2 (Mip-2) (Supporting Information Fig S1A). Conversely, increased expression of Irak1 by transient overexpression significantly enhanced the cellular response (Supporting Information Fig S1B). Enhanced Irak1 expression under certain clinical conditions such as oxygen deprivation might therefore result in innate immune hyper-responsiveness and contribute to immune-mediated tissue damage. A significant, time-dependent increase in Irak1 protein levels was observed during the course of oxygen deprivation (Fig 1A and Supporting Information Fig S1D). Induction of hypoxia was confirmed by enhanced expression of the hypoxia-inducible factor (Hif)-1α analysed by immunoblotting and immunostaining (Fig 1A and Supporting Information Fig S1C). Importantly, hypoxia induced a time-dependent increase in chemokine secretion in the presence of 1 ng/ml LPS, but not in the presence of the Tlr-independent stimulus phorbol myristate acetate (PMA, Fig 1B). Epithelial cells kept for 2 h under hypoxic conditions and subsequently stimulated under normoxic conditions exhibited a 10- to 50-fold increase in ligand sensitivity (Fig 1C and Supporting Information Fig S1E). Of note, oxygen deprivation did not lead to detectable cellular apoptosis during this time (Supporting Information Fig S1F) and the level of the Irak1 protein and innate immune hyper-responsiveness were fully reversible when oxygen deprivation ceased (Fig 1D).

### Hypoxia alters Irak1 ubiquitination and induces Senp1-mediated deSUMOylation

**Figure 2:**
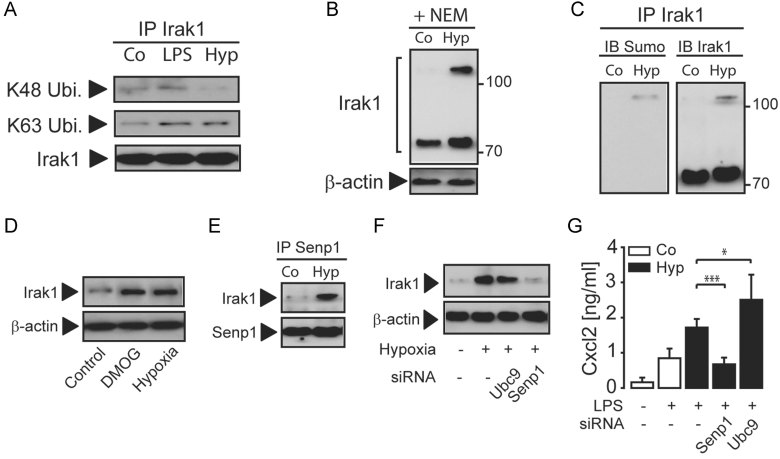
Hypoxia alters the ubiquitination pattern and induces Irak1 SUMOylation *Student's *t*-test *p* < 0.05, ***p < 0.001. Values are means ± SEM, from four independent experiments, *n* = 4/group. **A.** mIC_cl2_ cells were transfected with an ubiquitin-encoding plasmid and incubated in hypoxic chambers or treated with 10 ng/ml LPS for 2 h in the presence of the proteasome inhibitor MG132 (100 nM). Irak1 was immunoprecipitated, and K48 or K63 ubiquitination was individually detected by immunoblot.**B,C.** mIC_cl2_ cells were transfected with a plasmid-encoding haemagglutinin (HA)-tagged SUMO, left untreated (co.) or subjected to hypoxia in hypoxic chambers (Hyp.) for 2 h in the presence of 10 mM NEM and 100 nM MG132. Irak1 protein was detected by immunoblot (**B**), or (**C**) IRAK1 was immunoprecipitated, and SUMO-HA and Irak1 were detected by immunoblot.**D.** mIC_cl2_ cells were left untreated (control), treated with the hypoxia mimetic DMOG (1 mM), or incubated in hypoxic chambers for 2 h and Irak1 levels were determined by immunoblot.**E.** mIC_cl2_ cells were left untreated (co.) or incubated in hypoxic chambers (Hyp.) for 2 h, Senp1 was immunoprecipitated, and IRAK1 and SENP1 were detected by immunoblot.**F,G.** mIC_cl2_ cells were transfected with siRNA directed against *Senp1* or *Ubc9*, and incubated in hypoxic chambers for 2 h. (**F**) The level of Irak1 was determined by immunoblot, and (**G**) the amount of Cxcl2 secretion after stimulation with 1 ng/ml LPS for 6 h was quantified by ELISA. Hypoxia/Senp1 + LPS (0.331 ± 0.092) and Hypoxia/Ubc9 + LPS (2.564 ± 0.751) *versus* Hypoxia + LPS (1.481 ± 0.122), *p* = 5 × 10^−6^ and *p* = 0.02, respectively.

Oxygen deprivation did not affect *Irak1* mRNA suggesting a solely post-transcriptional mechanism of Irak1 protein upregulation in hypoxic IECs similar to the situation in endotoxin tolerance (Supporting Information Fig S2A; Chassin et al, 2010). Irak1 is ubiquitinated in order to induce both signal transduction and proteasomal degradation. Whereas K48 ubiquitin modification is a marker for non-signalling-prone Irak1 degradation, K63 ubiquitination facilitates signal transduction (Huang et al, 2005; Janssens and Beyaert, 2003; Newton et al, 2008). Interestingly, immunoprecipitation studies revealed that an increased fraction of K63 ubiquitin-modified Irak1 and a decreased fraction of K48 ubiquitin-modified Irak1 was detected in hypoxic m-IC_cl2_ cells compared to normoxic control cells, whereas both K48- and K63-ubiquitin-conjugated Irak1 was detected after LPS stimulation (Fig 2A and Supporting Information Fig S2B). These findings suggested that the reduction in the amount of K48 ubiquitin-conjugated Irak1 may contribute to the increased level of Irak1 protein in hypoxic cells. The enhanced level of K63-ubiquitin-conjugated Irak1 in turn might reflect a shift from silent Irak1 degradation to signal transduction-prone Irak1, causing a reduction of the signalling threshold upon receptor-ligand engagement.

Further analysis of m-IC_cl2_ cells overexpressing HA-tagged SUMO and incubated in the presence of the deubiquitination inhibitor *N*-ethylmaleimide (NEM) revealed a form of Irak1 with increased molecular size under hypoxic but not normoxic conditions (Fig 2B). This high-molecular weight form of Irak1 was also detected using an anti-SUMO antibody (Fig 2C) suggesting that Irak1 SUMOylation occurs under hypoxic conditions. This resembles the situation of the Hif-1α molecule that, under normoxic conditions, is proteasomally degraded after hydroxylation (Shao et al, 2004; Ulrich, 2007). Under hypoxic conditions, Ubc9-mediated SUMOylation and ubiquitination of Hif-1α facilitate proteasomal degradation. DeSUMOylation by the SUMO-specific protease (Senp)1, however, prevents the degradation and leads to enhanced protein levels in hypoxic cells (Cheng et al, 2007). Similarly, inhibition of hydroxylation with the cell permeable prolyl-4-hydroxylase inhibitor DMOG led to Irak1 accumulation under normoxic conditions (Fig 2D; Shao et al, 2004; Ulrich, 2007). Also, a direct molecular interaction was observed between Irak1 and Senp1 in hypoxic but not normoxic m-IC_cl2_ cells (Fig 2E). siRNA-mediated knockdown of Senp1 mRNA prevented both the hypoxia-induced accumulation of Irak1 protein (Fig 2F) and innate immune hyper-responsiveness (Fig 2G). In contrast, Irak1 accumulation and innate immune hyper-responsiveness were still observed under hypoxic conditions after siRNA-mediated silencing of the SUMO-conjugating enzyme *Ubc9*. Importantly, accumulation of Irak1 under hypoxic conditions was independent of Hif-1α or Hif-2α expression suggesting the presence of two similar but functionally independent processes (Supporting Information Fig S2C).

### I/R injury is associated with enhanced epithelial Irak1 and requires Tlr4- and Irak1-dependent signalling

**Figure 3:**
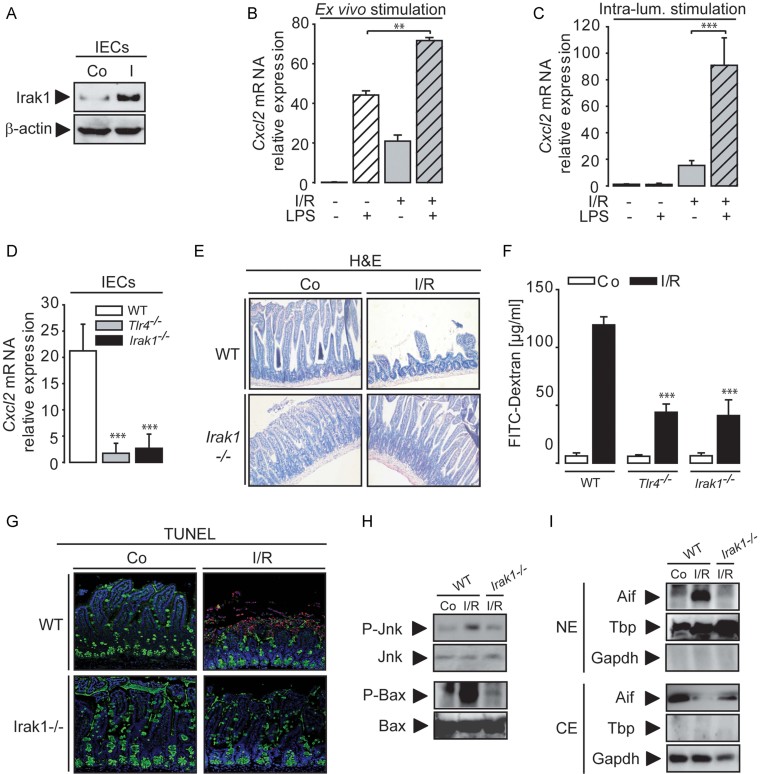
Ischemia-induced Irak1 accumulation leads to innate immune hyper-responsiveness and Tlr4- and Irak1-dependent tissue injury ***Student's *t*-test *p* < 0.001, **p < 0.01, compared to LPS-treated (**B**–**C**), WT (**D**), or WT I/R (**F**). Values are means ± SEM from 4 to 5 independent experiments. Magnification ×100. **A.** Wild-type mice (*n* = 12) were subjected to ischemia for 30 min (I), and IECs were isolated. Irak1 protein was determined by immunoblot.**B.** An ischemic segment and an unaffected control segment of the intestine were removed and incubated *ex vivo* for 2 h in the presence of 100 ng/ml LPS at 37°C. IECs were isolated and *Cxcl2* mRNA was quantified by real-time PCR. I/R + LPS (71.66 ± 1.56) *versus* I/R (44.20 ± 2.12), *p* = 0.004. *n* = 4 for each data point.**C.** After 30 min ischemia, 200 µl of a solution of 100 ng/ml LPS was injected intraluminally during a 1 h reperfusion period. IECs were then isolated, and *Cxcl2* mRNA was quantified by real-time RT-PCR. I/R + LPS (90.87 ± 20.70) *versus* I/R (15.32 ± 3.67), *p* = 3 × 10^−5^. *n* = 4 for each data point.**D–F.** Wild-type (WT), *Tlr4*^−/−^, and *Irak1*^−/−^ mice (*n* = 10 for each group) were subjected to I/R. (**D**) IECs were isolated, and *Cxcl2* mRNA was quantified by real-time RT-PCR. *Tlr4*^−/−^ (1.72 ± 1.91) and *Irak1*^−/−^ (2.67 ± 2.70) *versus* WT (21.23 ± 5.08), *p* = 5 × 10^−6^and *p* = 10^−5^, respectively. (**E**) H&E staining was performed using formalin-fixed tissue sections from I/R-treated or untreated (Co) intestinal segments. (**F**) Permeability of the intestinal barrier was measured by injecting 200 µl of a 25 mg/ml FITC-dextran solution into the intestinal lumen during ischemia (I/R) or into untreated control sections (Co). Subsequently, blood samples were collected and the fluorescence intensity was measured. *Tlr4*^−/−^ I/R (44.03 ± 7.06) and *Irak1*^−/−^ I/R (41.05 ± 13.64) *versus* WT I/R (119.95 ± 7.00), *p* = 5 × 10^−6^. *n* = 5 for each data point.**G.** TUNEL staining was performed using formalin-fixed tissue sections from I/R-treated or untreated (Co) intestinal segments.**H.** Expression of phospho-jnk (P-Jnk), Jnk, phosphor-Bax and Bax in IECs were assessed by Western blotting.**I.** The translocation of Aif was measured in nuclear extract (NE) and cytosolic extract (CE) of IECs (TATA binding protein Tbp and Gapdh expression were used as nuclear or cytosolic loading control respectively).

We then tested whether enhanced Irak1 expression also occurs in the intestinal epithelium of mice subjected to I/R *in vivo*. IECs isolated from an intestinal loop after transient interruption of the mesenteric blood flow for 30 min followed by restoration of the vascular flow for 60 min exhibited markedly increased Irak1 protein levels but no significant change in *Irak1* mRNA (Fig 3A and Supporting Information Fig S3A). Non-ischemic intestinal segments from the same animal were used as controls. Importantly, the increase in Irak1 was also associated with enhanced innate immune responsiveness. Significantly enhanced *Cxcl2* mRNA was found in high Irak1-expressing IECs isolated from post-ischemic wild-type intestinal segments exposed to LPS *in vitro* (Fig 3B and Supporting Information Fig S3B) or after intraluminal injection of LPS *in vivo* (Fig 3C). The level of *Cxcl2* mRNA was significantly enhanced in IECs isolated from post-ischemic intestinal segments of wild-type but not *Tlr4*^−/−^ or *Irak1*^−/−^ mice (Fig 3D). Also, the severity of tissue damage, mucosal translocation of intraluminally administered FITC dextran and the number of TUNEL-positive apoptotic IECs after I/R was markedly reduced in the absence of Tlr4 or Irak1 (Fig 3E–G and Supporting Information Fig S3C). I/R-induced epithelial cell death was associated with phosphorylation of c-jun N-terminal kinase (Jnk) and bcl2-associated x protein (Bax) and nuclear translocation of apoptosis-inducing factor (Aif) in isolated IECs from wild-type but not Irak1-deficient mice (Fig 3H and I). No detectable staining for active caspase 3 was detected (Supporting Information Fig S3D). These results are consistent with the previously established model of mitochondrion-dependent apoptosis through Jnk-mediated phosphorylation of Bax with subsequent release and nuclear translocation of Aif in the presence of enhanced levels of epithelial Irak1 protein (Kim et al, 2006; Takada et al, 2008).

### Induction of microRNA-146a lowers Irak1 and protects mice against intestinal I/R injury

**Figure 4:**
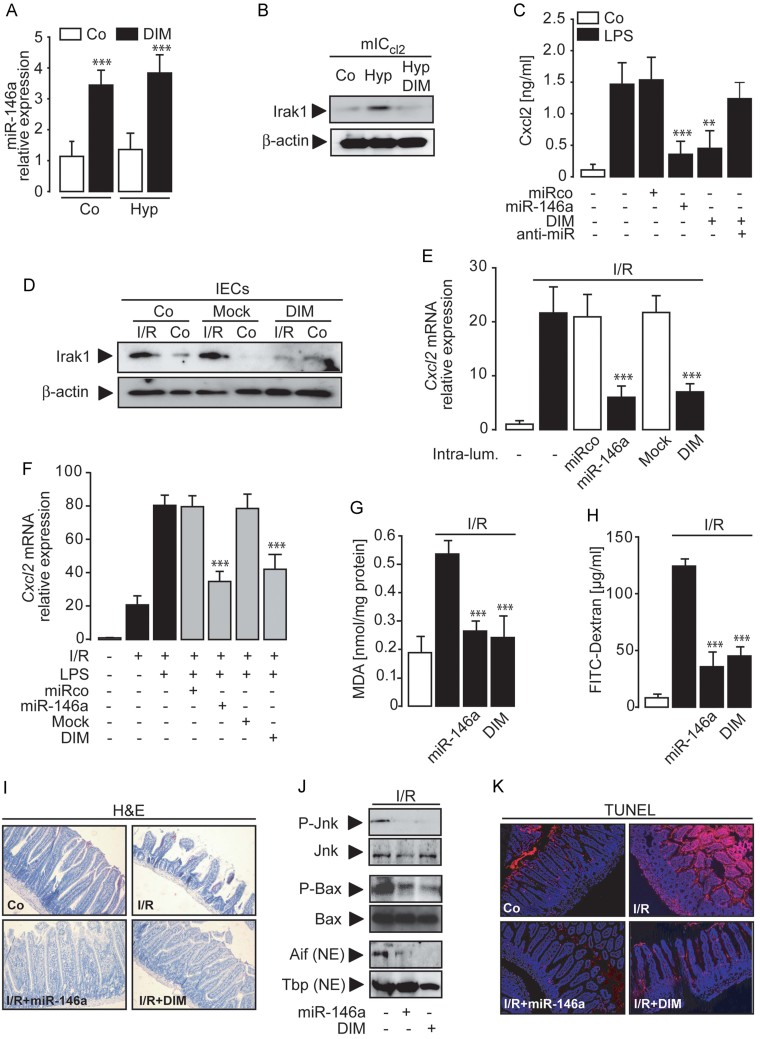
DIM-mediated induction of miR-146a diminishes the hypoxia-induced hyper-sensitivity towards LPS and protects against I/R injury in mice ***Student's *t*-test *p* < 0.001, **p < 0.01 compared to control (**A**), LPS-treated (**C**), I/R-treated (**E**, **G**, **H**), or I/R and LPS-treated (**F**). Values are means ± SEM from 4 to 5 separated experiments. Magnification ×100. **A,B.** mIC_cl2_ cells were incubated in hypoxic chambers for 2 h in the absence or presence of 25 µM diindolylmethane (DIM), and the levels of miR-146a (for each point, *n* = 4; Control: DIM 3.65 ± 0.58 *versus* Co 1.03 ± 0.64, *p* = 9 × 10^−4^; Hyp: DIM 3.99 ± 0.34 *versus* Co 1.21 ± 0.37, *p* = 3 × 10^−5^) (**A**) and Irak1 (**B**) were determined by real-time PCR and immunoblot, respectively.**C.** mIC_cl2_ cells were transfected with microRNA (miR)-146a mimic, an anti-miR-146a (anti-miR) or miR control (miRco), and/or treated with DIM and subjected to hypoxia for 2h. Cxcl2 mRNA was quantified by real-time RT-PCR after stimulating the cells with 1 ng/ml LPS for 6h (for each point, *n* = 4; miR-146a/LPS 0.440 ± 0.184 and DIM/LPS 0.475 ± 0.194 *versus* LPS 1.1516 ± 0.363, p = 10^−3^ and *p* = 0.002, respectively).**D.** 200 µl of 25 µM DIM or the solvent control DMSO (Mock) was intraluminally injected into the small intestine of WT mice (*n* = 10 for each group). Subsequently, the intestinal tissue was subjected to ischemia for 30 min followed by reperfusion for 1 h. IECs were isolated and Irak1 protein was determined by immunoblotting.**E.** 100 nM miR-146a mimic, 100 nM miR control (miRco), 25 µM DIM or the solvent control (Mock) was intraluminally injected into the small intestine of WT mice (*n* = 10 for each group). Subsequently, the intestinal tissue was subjected to ischemia for 30 min, followed by reperfusion for 1 h. IECs were isolated and Cxcl2 mRNA was quantified by real-time PCR. miR-146a/IR 3.17 ± 1.17 and DIM/IR 3.59 ± 0.85 *versus* IR 21.24 ± 2.20, *p* = 6 × 10^−6^ and *p* = 5 × 10^−6^, respectively.**F.** Epithelial Cxcl2 mRNA in response to intraluminal injection of 100 ng/ml LPS during the reperfusion period after pretreatment with miRco, miR-146a, DIM or Mock was determined by quantitative PCR. miR-146a/IR/LPS 34.90 ± 5.98 and DIM/IR/LPS 42.19 ± 8.88 *versus* IR/LPS 80.51 ± 6.09, *p* = 4 × 10^−5^ and *p* = 4 × 10^−4^, respectively. For each data point, *n* = 5.**G.** Lipid peroxidation, used as an indirect index of the oxidative injury induced by the reactive oxygen species, was determined by measuring the malonedialdehyde (MDA) concentration with the thiobarbiturate reaction. miR-146a/IR 0.2633 ± 0.0198 and DIM/IR 0.2467 ± 0.715 *versus* IR 0.5304 ± 0.0446, *p* = 3 × 10^−5^ and *p* = 5 × 10^−4^, respectively. For each data point, *n* = 5.**H.** The permeability of the intestinal barrier was measured by intraluminal injection of 200 µl 25 mg/ml FITC-dextran during the ischemia period after pretreatment with miRco, miR-146a, DIM or Mock. Blood samples were collected after reperfusion, and the fluorescence intensity was determined. miR-146a/IR 35.66 ± 12.99 and DIM/IR 45.16 ± 7.99 *versus* IR 122.61 ± 3.51, *p* = 9 × 10^−4^ and *p* = 2 × 10^−4^, respectively. For each data point, *n* = 4.**I.** H&E staining of small intestinal tissue sections left untreated (co) or after I/R with or without pretreatment with miR-146a or DIM.**J.** Levels of phospho-Jnk (P-Jnk), phospho-Bax (P-Bax) and nuclear Aif was determined by immunoblot.**K.** TUNEL staining of small intestinal tissue sections left untreated (co) or after I/R with or without pretreatment with miR-146a or DIM.

We and others have previously demonstrated that Irak1 *in vivo* is regulated on the post-transcriptional level by miR-146a (Boldin et al, 2011; Carthew and Sontheimer, 2009; Chassin et al, 2010). miR-146a in turn was recently shown to be induced by the naturally occurring substance 3,3′-diinodolylmethane (DIM; Li et al, 2010). In accordance, a significant increase in miR-146a was found in m-IC_cl2_ cells after the administration of DIM under both normoxic and hypoxic conditions (Fig 4A). DIM didn't have any effect on the expression of additional miRs known to be involved in the regulation of inflammation or I/R injury such as Let-7a, miR-21, miR-155 and miR-29a in our model (Supporting Information Fig S4B–E). Importantly, DIM also reversed the effect of hypoxia on epithelial Irak1 protein expression (Fig 4B). Furthermore, the increased secretion of Cxcl2 by hypoxic m-IC_cl2_ cells decreased after transfection of miR-146a or incubation with DIM but not in the presence of control microRNA (miRco) (Fig 4C). Also *in vivo*, the enhancement of Irak1 protein levels after I/R was abolished following the intraluminal injection of DIM but not of the solvent control (mock) (Fig 4D). Intramural injection of DIM or miR-146a also significantly reduced the *Cxcl2* mRNA levels after I/R (Fig 4E) and diminished the I/R-mediated hyper-responsiveness of the intestinal epithelium to LPS (Fig 4F). Epithelial uptake of microRNA after luminal exposure was confirmed using fluorescently conjugated miR control (Mimic co; Supporting Information Fig S4A). Finally, lipid oxidation as a consequence of oxidative injury, enhanced translocation of FITC dextran through the intestinal mucosa, intestinal tissue damage, Jnk and Bax phosphorylation, Aif nuclear expression and epithelial apoptosis were all significantly lower in post-ischemic intestinal segments after the intraluminal administration of miR-146a or DIM than in mock-treated, post-ischemic tissue segments (Fig 4G–K).

### DIM lowers Irak1 levels and reduces hypoxia-induced innate immune hyper-responsiveness in human intestinal tissue

**Figure 5:**
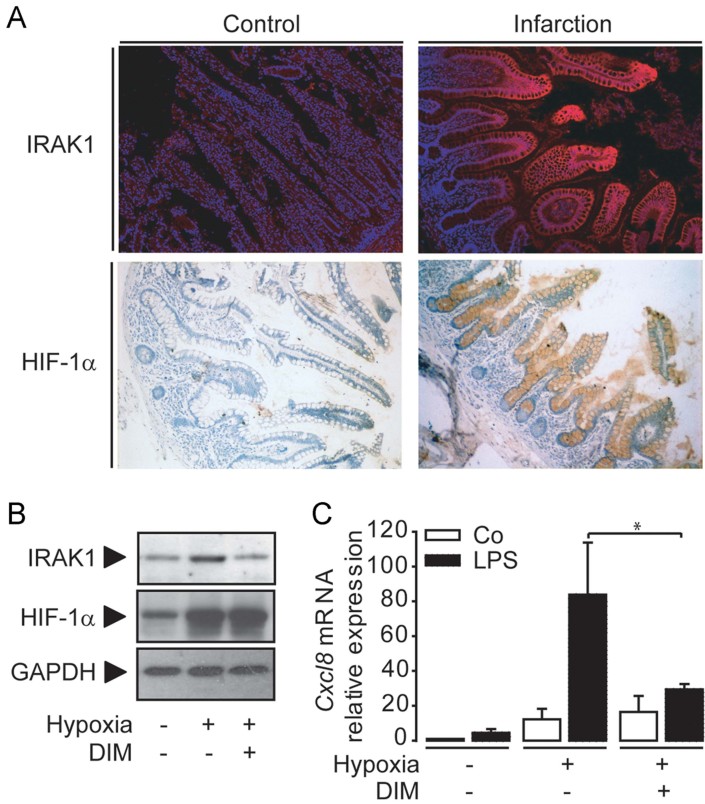
DIM-mediated decrease in IRAK1 reduces hypoxia-induced innate immune hyper-responsiveness in human intestinal mucosa **A.** Immunohistology for IRAK1 and HIF-1α in tissue sections of human small intestine obtained from normoxic controls or patients suffering from mesenteric infarction. Magnification ×100.**B,C.** Human small intestinal biopsies (*n* = 5) were left untreated or subjected to hypoxia for 2 h in the absence or presence of DIM. (**B**) IRAK1 and HIF-1α was determined by immunoblot and (**C**) LPS susceptibility was analysed by quantitative RT-PCR for Cxcl8 RNA after incubating for 6 h in the absence or presence of 10 ng/ml LPS. Hypoxia/DIM 29.45 ± 3.04 *versus* hypoxia 83.88 ± 29.90, *p* = 0.02. For each data point, *n* = 5.*Student's *t*-test *p* < 0.05 between groups. Values are means ± SEM from five separated experiments.

Next, human intestinal tissue samples from patients suffering from intestinal infarction and from healthy controls were stained for HIF-1α and IRAK1. Intense positive IRAK1 immunostaining was noted in the human intestinal mucosa from patients after mesenteric infarction but not in that from control subjects (Fig 5A, upper panel). Mucosal ischemia was confirmed by strong HIF-1α immunostaining (Fig 5A, lower panels). Also, incubation of human intestinal tissues *ex vivo* under hypoxic conditions for 2 h induced enhanced IRAK1 levels. Exposure of human tissue samples under hypoxic or normoxic conditions to 25 µM DIM led to significantly greater expression of miR-146a than in mock-treated tissue samples (Supporting Information Fig S5). Interestingly, the increase in IRAK1 protein was prevented in the presence of the miR-146a-inducing agent DIM (Fig 5B). Importantly, the increased miR-146a and reduced IRAK1 levels observed after treatment with DIM led to significantly diminished *Cxcl8* mRNA expression of human biopsies after stimulation with LPS (Fig 5C).

## DISCUSSION

The intestinal mucosa is particularly susceptible to ischemic insult. Mesenteric artery occlusion, aortic aneurysm repair, hypovolemic or cardiogenic shock, transplantation and premature birth are all associated with reduced tissue perfusion and frequently lead to hypoxic tissue damage causing significant morbidity and mortality. Mucosal ischemia resulting from fibrosis-induced microvascular insufficiency has also been observed in chronic inflammatory bowel disease and might contribute to the progression of intestinal lesions (Colgan and Taylor, 2010; Wakefield et al, 1989). A firm association between I/R injury and Tlr activation has been established, based mainly on improved outcomes in animals deficient in Tlr2, 4 or 9, all of which signal through the adaptor molecule MyD88 and Irak1 to induce NF-κB activation (Bamboat et al, 2010; Ellett et al, 2009; Moses et al, 2009; Suzuki et al, 2008; Zanotti et al, 2009). Uncontrolled innate immune activation of the enteric epithelium by the enteric microbiota is thought to contribute significantly to intestinal I/R injury (Chen et al, 2003; Yoshiya et al, 2011; Zou et al, 2003). Administering NF-κB blocking agents, however, did not significantly improve the clinical outcome (Chen et al, 2003; Zou et al, 2003). These findings thus suggest that it is not innate immune signalling as such, but rather hyper-responsiveness of the innate immune system that constitutes the underlying mechanism of I/R injury. The findings of the present study demonstrate that enhanced levels of the Irak1 protein at the intestinal epithelium following ischemia contribute to the Tlr-mediated tissue damage associated with I/R injury.

In response to ligand engagement, Irak1 is recruited to the Tlr/MyD88/Irak4 receptor complex. Irak1 phosphorylation then allows binding of tumour necrosis factor (Tnf) receptor-associated factor (Traf)6 (Gottipati et al, 2008; Thomas et al, 1999). Ubiquitination of Irak1 by Lys63-linked (K63) polyubiquitin is subsequently required to promote downstream signalling via the NF-κB essential modifier (Nemo) and inhibitor of kappa-B kinase (Ikk) (Windheim et al, 2008). On the other hand, decoration of Irak1 by Lys48-linked ubiquitin chains mediates proteasomal degradation and contributes to the termination of cell activation (Newton et al, 2008). In accordance with a protective role of ubiquitin-mediated protein degradation, enhanced proteasomal function in mice overexpressing the proteasomal subunit PA28α was shown to protect from I/R injury (Li et al, 2011). Recent evidence suggests that SUMOylation is functionally closely interlinked with other post-transcriptional modifications including ubiquitination and thus contributes to an integrated regulatory control system (Gareau and Lima, 2010). Our results demonstrate for the first time that SUMOylation of Irak1 occurs under hypoxic conditions. Senp1-mediated deSUMOylation prevented ubiquitin-mediated degradation and increased the level of Irak1 protein in a manner similar to the recently discovered mechanism facilitating the stabilization of Hif-1α or IκBα under oxygen restriction (Melvin et al, 2011; Ulrich, 2007). The increased formation of K63 ubiquitin together with SUMOylation-mediated stabilization of Irak1 under hypoxic conditions thus indicates an enhanced Il-1R/Tlr-mediated signal transduction capacity. Increased epithelial Irak1 expression corresponded with the observed post-ischemic innate immune hyper-responsiveness and was associated with stimulation of Jnk-mediated signal transduction, phosphorylation of Bax, release and nuclear translocation of the apoptosis promoting Aif, epithelial apoptosis and mucosal barrier disruption.

Similar to oxygen deprivation, administration of the hydroxylase inhibitor DMOG under normoxic conditions enhanced epithelial Irak1 protein levels both *in vitro* and *in vivo* when injected in the intestinal lumen consistent with the involvement of hydrolases in the regulation. In accordance, enhanced baseline levels of epithelial *Cxcl2* mRNA expression were noted (unpublished observation). Previous studies have reported on an ecto-5′ nucleotidase (CD73)- and A2B adenosine receptor (A2BAR)-mediated protective effect of systemic DMOG administration on Hif-1α-mediated I/R injury (Hart et al, 2011) and other models of intestinal mucosal inflammation (Cummins et al, 2008; Hindryckx et al, 2010; Robinson et al, 2008). Own studies on local DMOG administration under ischemic conditions revealed not significant influence on epithelial lipid oxidation, mucosal barrier disruption and tissue damage (unpublished observation) possibly due a mixed CD73- and A2BAR-mediated protective and Irak1-induced aggravating effect. Together, these results are in accordance with the hypothesis that Hif-1α and Irak1 act as two similar but functionally independent pathways downstream of tissue oxygen deprivation.

In addition, our results indicate a novel strategy to prevent I/R tissue injury. Irak1 protein expression is controlled by microRNA (miR)-146a mediated translational repression, providing a negative regulatory circuit of Il-1R and Tlr signalling in IECs (Boldin et al, 2011; Chassin et al, 2010; Gottipati et al, 2008). Our results show that local administration of miR-146a itself or the miR-146a inducing agent DIM (Li et al, 2010) during the ischemic period is able to prevent the cellular accumulation of Irak1 protein, restrict immune hyper-responsiveness and prevent I/R-induced tissue damage in both an *in vivo* murine I/R model and in human intestinal tissue samples incubated under hypoxic conditions. Interestingly, enhanced expression of endogenous miR-146a has recently been observed late during renal I/R and might reflect an endogenous inhibitory loop (Godwin et al, 2010). Our results might be of particular interest in the context of previous results. Although pharmacological impairment of the activation of Tlr4 or of NF-κB decreased the cellular response to I/R injury (Suzuki et al, 2008; Wu et al, 2009), complete blockade of Ikk or NF-κB was detrimental and aggravated epithelial I/R-mediated apoptosis and tissue damage (Chen et al, 2003; Zou et al, 2003). Inhibiting innate immune hyper-responsiveness rather than completely blocking receptor signalling and homeostatic innate immune-mediated signalling required for cell survival and tissue repair might, therefore, offer a promising strategy (Shulzhenko et al, 2011). This strategy functionally resembles ischemic preconditioning, which involves briefly interrupting the vascular blood supply in order to improve the tissue's tolerance of subsequent prolonged ischemia. Even very short periods of ischemia induce significant NF-κB activity, and might therefore enhance the NF-κB-regulated miR-146a (Chassin et al, 2010; Ferencz et al, 2006; Godwin et al, 2010; Taganov et al, 2006).

In conclusion, we have identified the underlying mechanism of I/R-induced innate immune hyper-responsiveness of IECs demonstrating that hypoxia reduces the K48 ubiquitinated degradation-prone fraction of epithelial Irak1 and stimulates Senp1-mediated Irak1 deSUMOylation. Enhanced Irak1 levels lower the threshold of microbial innate immune receptor stimulation, enhance the secretion of proinflammatory chemokines and cause I/R-induced barrier disruption and mucosal tissue injury. We further provide conclusive evidence that administration of miR-146a or pharmacological induction of epithelial miR-146a expression diminishes post-ischemic Irak1 protein accumulation, innate immune hyper-responsiveness and tissue damage. Pharmacological modulation of miR-146a may therefore represent a new way to reduce the tissue damage and organ dysfunction associated with I/R.

## MATERIALS AND METHODS

### Reagents

Microencapsulated BioResponse diindolylmethase (DIM) was provided by Michael A. Zeligs (BioResponse, LLC, Boulder, CO, USA). *Escherichia coli* K12 D31m4 LPS was obtained from List Biological Laboratories and phorbol 12-myristate 13-acetate (PMA), MG132, dimethyloxalylglycine (DMOG) and NEM from Sigma–Aldrich. Endotoxin contamination was excluded using the chromogenic QCL-1000 limulus amebocyte lysate assay (BioWhittaker).

### Cell culture assays

m-IC_cl2_ cells were cultured as described (Bens et al, 1996; Chassin et al, 2010; Lotz et al, 2006). RNA silencing was performed with predesigned siRNAs (Flexitubes siRNA, QIAGEN) for *Tlr4*, *Irak1*, *Senp1* and *Ubc9* and a universal negative control (final concentration 10 nM). For miR transfection, a miR-146a miRIDIAN mimic and a Cy3-labelled miRIDIAN mimic control (Dharmacon RNAi Technologies) at a final concentration of 100 nM was used (Chassin et al, 2010). For overexpression, m-IC_cl2_ cells were transfected with 1 µg of pDEST515 control vector or pDEST515 IRAK1 wt construct (generously provided by Jonathan D. Ashwell, National Institutes of Health, Bethesda, USA) using Lipofectamin 2000 (Invitrogen). The same method was used to transfect the plasmids pUbiquitin and pSUMO-HA (obtained from Kristina Lindsten, Karolinska Institute, Stockholm, Sweden). Hypoxia was induced *in vitro* by overlaying m-IC_cl2_ cells with mineral oil as described (Mkaddem et al, 2010; Vanheel et al, 1989; Wu et al, 2007), or by incubating the cells in anaerobic chambers. For hypoxic treatment in chambers, cells were placed in an airtight container (Thermo Scientific) and maintained at 37°C in humidified atmosphere containing 1% O_2_ and 10% CO_2_. O_2_ and CO_2_ levels were verified with a Datex-Ohmeda Capnomac Ultima monitor. Media were equilibrated before use for 24 h to the required oxygen level in the hypoxia workstation in the same conditions. Conventional control cultures kept at 37°C in ambient air (21% O_2_) in a standard incubator with 95% humidified atmosphere and 5% CO_2_. Cell viability was determined by flow cytometry using FITC-conjugated annexin V (EMELCA Biosciences).

### Mice

C57BL/6 mice were purchased from Charles River Breeding Laboratories, housed under specific pathogen-free conditions, and treated in accordance with the local animal protection legislation (Niedersächsisches Landesamt für Verbraucherschutz und Lebensmittelsicherheit Oldenburg). *Tlr4*-deficient (*Tlr4*^−/−^) C57BL/10ScN mice were generously provided by Marina Freudenberg (Max Planck-Institute of Immunobiology, Freiburg, Germany) and *Irak1*-deficient (*Irak*^−/−^) C57BL/6 mice by J. Thomas (University of Texas Southwestern Medical Center, Dallas, TX, USA) and Anne Krug (University Clinic, Technical University Munich, Munich, Germany). For I/R and *ex vivo* stimulation methods, see Supporting Information.

### Human intestinal biopsies

The study was approved by the local Ethics Committee at Hannover Medical School (Nr. 788), and samples were obtained after informed written consent. A total of 10 mucosal tissue samples were collected from apparently healthy areas of the terminal ileum of five patients (age 31–50) with a history of inflammatory bowel disease (ulcerative colitis and Crohn's disease), surgical resection of a rectum carcinoma, polyposis coli or partial hypertensive colonopathy undergoing an elective control endoscopy. Tissue samples were immediately transferred to the laboratory in cold sterile 0.9% NaCl solution. Biopsies were subjected to hypoxia as described. After 2 h, samples were washed 3 times with prewarmed 0.9% NaCl, incubated for 2 h in cell culture medium in the absence or presence of 100 ng/ml LPS and analysed. Immunostaining for HIF-1α and IRAK1 was performed on tissue sections from patients undergoing tissue resection following mesenteric infarction or cancer.

### *In vivo* intestinal permeability assay

Prior to the induction of ischemia, 200 µl PBS containing 25 mg/ml 4.4-kDa fluorescein isothiocyanate (FITC)-dextran (FD-4; Sigma–Aldrich) was intraluminally administered. After the reperfusion period, mice were euthanized, and a blood sample (100 µl) was obtained by cardiac puncture. The concentration of FITC-dextran in plasma was measured using a fluorescence spectrophotometer (Vector).

### Lipid peroxidation assay

Lipid peroxidation was assessed by using the Lipid Peroxidation (MDA) Assay Kit (Biovision) according to the manufacturer instructions. Briefly, after isolation, IECs were homogenized on ice in the MDA Lysis Buffer, then TBA solution was added into each vial containing standard and sample. After incubation at 95°C for 60 min and cooling in an ice bath for 10 min, the absorbance was read at 532 nm.

**The paper explained**

PROBLEM:

Ischemia/reperfusion (I/R) injury is observed in a variety of clinical conditions such as vascular occlusion, haemorrhagic shock, trauma or following solid organ transplantation and associated with high morbidity and mortality. I/R in the intestine has additionally been implicated in the pathogenesis of necrotizing enterocolitis in preterm delivered neonates. Innate immune hyper-responsiveness mediated by enhanced Tlr signalling has been identified in the pathogenesis of I/R injury but the underlying molecular mechanisms have remained ill-defined and novel prophylactic and therapeutic strategies are needed.

RESULTS:

Using a mouse model of intestinal I/R injury and human intestinal mucosal biopsies, we observed enhanced protein expression of the essential Tlr signalling molecule Irak1 in ischemic epithelial cells associated with a striking increase in the responsiveness to innate immune stimulation. Enhanced Irak1 expression was associated with increased ligand responsiveness, chemokine secretion, epithelial apoptosis, mucosal barrier disruption and tissue destruction in an I/R model whereas Irak1-deficient mice were protected from ischemia-mediated tissue damage. Irak1 protein accumulation under hypoxic conditions was caused by changes in the ubiquitination pattern and Ubc9-mediated transient SUMOylation of Irak1. Importantly, administration of miR-146a or the miR-146a-inducing agent DIM controlled epithelial Irak1 protein levels in mouse and human mucosal tissue by translational repression and protected from I/R injury.

IMPACT:

We identify Irak1 protein as a major regulator of Tlr-mediated innate immune responsiveness in IECs and show that administration or pharmacological induction of miR-146a represents a new strategy to control innate immune hyper-responsiveness and reduce tissue damage after transient hypoxia or I/R.

### Quantitative mRNA measurements

Quantitative real-time PCR was performed as previously described (Chassin et al, 2010). TaqMan gene expression assays for murine hypoxanthine phosphoribosyltransferase (*Hprt*), *Cxcl2* and *Irak1* (Applied Biosystems) and specific TaqMan hybridization probes to quantify miR-146a, miR-21, let-7a, miR-155 and miR-29b (TaqMan microRNA assay, Applied Biosystems) were used. The small RNA snoRNA202 was amplified as internal control (TaqMan snoRNA202 assay, Applied Biosystems). Analyses were performed with a CFX96 Real-Time PCR Detection System (Bio-Rad). Each sample was amplified in duplicate, and normalized *versus* the endogenous control. Results were calculated using the 2-ΔΔ*C*_t_ method, and are presented as the fold induction of the target gene transcript under stimulated *versus* unstimulated conditions.

### Immunoblot, immunoprecipitation and ELISA

Immunoblotting was performed as recently described (Chassin et al, 2010). Antibodies against mouse IRAK1, SENP1 (both from Santa Cruz Biotechnology), human IRAK1, GAPDH, HIF-2α, JNK, phospho-JNK, Bax, AIF, (from Cell Signalling), TBP, phospho-Bax (both from Abcam), Lys48 (K48)-specific ubiquitin, Lys63 (K63)-specific ubiquitin (both from Millipore), HIF-1α (Novus Biologicals), SUMO and β-actin (both from Sigma–Aldrich) were used in combination with peroxidase (PO)-labelled goat anti-mouse, goat anti-rat or goat anti-rabbit secondary antibodies (Jackson ImmunoResearch). Immunoprecipitations were performed with the protein A Immunoprecipitation Kit (Roche Applied Science). MIP-2 in cell culture supernatant was determined by ELISA (Nordic Biosite; Hornef et al, 2002).

### Immunostaining

Staining for TUNEL (Roche Diagnostics), Caspase 3 (Cell Signaling Technology), HIF-1α (Novus biologicals) and IRAK1 (Cell Signaling) was performed as recommended. A biotinylated goat anti-mouse IgG and an AF555-conjugated donkey anti-rabbit antibody (both from Jackson Immunoresearch Laboratories) were used as secondary antibodies. Paraffin sections were stained with H&E using a standard protocol. Counterstaining was performed with fluorescein-conjugated wheat germ agglutinin (WGA, 1:2000, Vector) or MFP488 phalloidin (1:50, MoBiTec) as indicated. Slides were mounted in DAPI containing Vectashield (Vector) and visualized with an ApoTome-equipped Axioplan 2 microscope connected to an AxioCam Mr digital Camera (Carl Zeiss MicroImaging).

### Statistical analysis

Results are expressed as means ± SD, and are representative of at least three independent experiments for each of the experimental conditions tested. Differences were analysed with the unpaired Student's *t* test. *p* < 0.05 was considered significant.

For more detailed Materials and Methods see the Supporting Information.

## Supplementary material

Detailed facts of importance to specialist readers are published as ”Supporting Information”. Such documents are peer-reviewed, but not copy-edited or typeset. They are made available as submitted by the authors.
