# Supplementary material for: MicroRNA-146a-mediated downregulation of IRAK1 protects mouse and human small intestine against ischemia/reperfusion injury
Source: EMBO Mol Med. 2012 Nov 9;4(12):1308–19. doi: 10.1002/emmm.201201298 (PMC3531605; doi:10.1002/emmm.201201298)
Supplement: Supplementary file 1 [file emmm0004-1308-SD1.pdf]

EMBO
Molecular Medicine

Manuscript EMM-2012-01298

MicroRNA-146a-Mediated Downregulation of Irak1 Protects Mouse and Human Small Intestine against Ischemia/Reperfusion Injury

Cécilia Chassin, Cordelia Hempel, Silvia Stockinger, Aline Dupont, Joachim F. Kübler, Jochen Wedemeyer, Alain Vandewalle, Mathias W. Hornef

Corresponding author: Cécilia Chassin, Hannover Medical School

Review timeline:

Submission date:	16 February 2012
Editorial Decision:	25 March 2012
Revision received:	02 July 2012
Editorial Decision:	25 July 2012
Revision received:	21 September 2012
Accepted:	25 September 2012

Transaction Report:

(Note: With the exception of the correction of typographical or spelling errors that could be a source of ambiguity, letters and reports are not edited. The original formatting of letters and referee reports may not be reflected in this compilation.)

1st Editorial Decision 25 March 2012

Thank you for the submission of your manuscript "MicroRNA-146a as Therapeutic Target to Prevent Intestinal Ischemia/Reperfusion Injury" to EMBO Molecular Medicine. We have now heard back from the three referees whom we asked to evaluate your manuscript. You will see that they find the topic of your manuscript potentially interesting. However, they also raise significant concerns on the study, which should be addressed in a major revision of the manuscript.

In particular, reviewers #1 and #2 highlight that the investigation of the therapeutic aspects of the study should be extended and make specific suggestions for that. Importantly, Reviewer #1 highlights that it is crucial to use hypoxic chambers instead of mineral oil to mimic hypoxia in vitro. Referee #3 feels that the mechanistic insight of the study is rather limited and suggest alternative paths that could be considered to strenghen the paper.

On a more editorial note, EMBO Molecular Medicine requires that the description of all reported data that includes statistical testing must state the name of the statistical test used to generate error bars and P values, the number (n) of independent experiments underlying each data point (not replicate measures of one sample), and the actual P value for each test (not merely 'significant' or 'P > 0.05') (please see http://onlinelibrary.wiley.com/journal/10.1002/(ISSN)1757-4684/homepage/ForAuthors.html#data2 for more information). Please also include a Table of Contents as the first page of your Supporting Information.

Given the balance of these evaluations, we feel that we can consider a revision of your manuscript if

you can convincingly address the issues that have been raised within the space and time constraints outlined below.

Revised manuscripts should be submitted within three months of a request for revision. They will otherwise be treated as new submissions, unless arranged otherwise with the editor.

I look forward to seeing a revised form of your manuscript as soon as possible.

Yours sincerely,

Editor
EMBO Molecular Medicine

***** Reviewer's comments *****

Referee #1 (Comments on Novelty/Model System):

The in vivo studies are strong but the use of mineral oil immersion to mimic hypoxia in vitro is inadequate. The authors have gone to some degree to overcome this by using hypoxia / anaerobic chambers but this needs further work.

Referee #1 (Other Remarks):

SUMMARY:
This is an interesting manuscript which addresses a clinically important question. However some key issues need to be resolved. Firstly, the authors have previously demonstrated that IRAK-1 suppression is protective in bacteria-induced damage in the neonatal gut and that miR-146a targets IRAK-1 to provide protection. (Chassin et al 2010; Lotz et al. 2006). Therefore, some further in vivo experimentation is required to provide the necessary novelty to justify publication in EMBO Molecular Medicine. Secondly, from a technical point of view, I do not accept the use of mineral oil overlay as a mimic of hypoxia. In my opinion, these experiments should be done using a controlled environment chamber as is the current state-of-the-art in the field. Any in vitro work demonstrating the effects of hypoxia should use hypoxia chambers (and not oil immersion). Thirdly, the authors show that the hypoxia mimetic DMOG also increases IRAK1 expression in vitro. This would predict that DMOG would exacerbate IR injury whereas DMOG has previously been shown to be protective in intestinal IR injury (Hart et al J. Immunol 2011). Therefore the authors should test the effects of hydroxylase inhibition with DMOG in their model.
REVIEW:
In this manuscript, Chassin et al. present data supporting a model which proposes that ischemia-reperfusion-induced IRAK-1 posttranscriptional upregulation leads to TLR-dependent immune hyper-reactivity associated with reperfusion injury in intestinal epithelial cells. The authors also suggest that delivery of miRNA 146a may represent a therapeutic intervention strategy in IRI. Understanding the immunologic basis and developing new therapeutics for reperfusion injury is an interesting and important area of research. The current manuscript contains some interesting data (especially the in vivo studies). However some significant experimental and methodological issues exist which need to be addressed.
Major Issues:
1) The use of mineral oil to elicit hypoxia in cells in culture is not, in my opinion, a valid technique. It is not possible to ascribe the effects of mineral oil to hypoxia alone and there is no appropriate negative control to account for the physical effects of the oil. In this study, anaerobic chambers were also used and some of this data is shown in the supplementary information, however it is not clearly detailed which experiments used anaerobic chambers and which experiments used mineral oil. In my opinion, the primary data reported should only be those generated using hypoxia/anaerobic chambers with an appropriately controlled environment and defined oxygen levels. Negative control cells should be cultured in an incubator with the same conditions (e.g. temperature, CO_2) and a lower oxygen level.

2) In figure S2B, the authors need to show that the knockdown of HIF-1 and HIF-2 was successful.

3) The authors demonstrate that the hydroxylase inhibitor DMOG also upregulates IRAK-1

(curiously, this appears to be independent of HIF). As such, DMOG would be expected to exacerbate the effects of IR injury. Previous reports indicate that this is not the case and in fact that DMOG is protective (Hart et al., J. Immunol 2011). This begs the question, what is the impact of hydroxylase inhibition (e.g. DMOG administration) on IR injury in this model?

4) Are the effects of DIM specific for miR-146a in this model? Are other miRNAs not induced? The authors need to demonstrate this by examining the effects of DIM on other miRNAs.

Minor Issues:

1) Abstract line 1 typo- "damage" (not damages).

2) The labeling of Fig S1C and S1D do not match between the figure and the text.

3) In figure 2, was hypoxia induced using mineral oil or a hypoxia/anaerobic chamber? The experiments described here should be using the chamber system.

Referee #2 (Comments on Novelty/Model System):

Of potential interest. However, the manuscript need further experiments to support claims of therapeutic effects of micrRNA146a or DIM before accepting publication

Referee #2 (Other Remarks):

In this manuscript the authors initially seek to establish and characterize the involvement of IRAK1 as part of the TLR response, in tissue damage following I/R insult in the intestinal cells, mouse intestines and human intestinal explants. This part of the manuscript is very interesting, convincing and thorough, and the manuscript is very well written. The authors subsequently focus on therapeutic strategies using (miR)-146a to address I/R injury via stabilization of IRAK1, which is of potential interest. However the observation is not novel as the authors themselves and others have already shown IRAK1 regulation by (miR)- 146a. As the scope of the manuscript is intended to be therapeutic, the following issues would be needed in order to strengthen the therapeutic effect of (miR)-146a and DIM on I/R injury.

Specific comments:

The major concern of this manuscript, given the title presented - "the therapeutic effect of (miR)-146a or DIM on I/R injury", is that it is not adequately substantiated and in part inconclusive. More in vivo experiments using mice, assessing dose dependant effect of DIM or (miR)- 146a, oral administration (gavage) of DIM post I/R injury i,e evaluating its therapeutic effect (post injury) needs to be analyzed.
In classical I/R injury, reactive oxygen species are thought to be the major cause of tissue damage, furthermore IRAK1 has also been shown to induce ROS generation. It would be valuable in this context to assess the levels of ROS like lipid peroxidation or reduced glutathione (GSH) or hydroxyl radicals, pre and post DIM treatment in mice.
The Tunel assay immunostainings especially in figures 3F and 4F are less convincing enough and needs to be replaced by better quality images with higher magnification and resolution. Moreover, the authors appear not to consider the possibility of necrosis that is quite likely to be taking place in parallel to apoptosis during I/R. It is equally possible that there could be more of necrosis than apoptosis under these circumstances. Since Tunel assay fails to discriminate between the two processes of cell death, it may be further substantiated with assays specific for apoptosis or necrosis and this issue should be adequately discussed.
The sentence in results section "A significant, time-dependent increase in IRAK1 was observed during the course of oxygen deprivation using two different models (Fig. 1A and S1C)." is misleading as in both the figures the same model "oil overlay" has been used. Moreover, S1C shows no time dependence at all.

In the results section, in line "Induction of hypoxia was confirmed by enhanced expression of the hypoxia-inducible factor (HIF)-1á analyzed by immuno-blotting and immuno-staining (Fig. 1A and S1D)" either of the mentioned figures does not show any immuno-staining. These discrepancies between results and figures are confusing and needs to be corrected.
In the sentence "Interestingly, the increase in IRAK1 protein was prevented in the presence of the miR-146a inducing agent, DIM (Fig. S5B).", the mentioned figure S5B does not exist rather should be 5B. The authors need to properly revise the manuscript in order to correct this and other similar mistakes.

Referee #3 (Other Remarks):

In this manuscript the authors show that:

1. Hypoxia in IECs in vitro leads to increased IRAK1 levels, increased MIP2 expression and sensitization to LPS
2. Hypoxia in IECs in vitro leads to an alteration of the IRAK1 K48/K63 ubiquitination ratio and induces IRAK1 sumoylation
3. IRAK1 and MIP2 are increased in I/R injury. IRAK1-/- mice show attenuated I/R injury pathology, decreased gut permeability and apoptosis
4. Induction of miR146a reduces IRAK1 and MIP2 levels in-vitro and in-vivo and leads to attenuated I/R injury, decreased gut permeability and apoptosis
5. Increased IRAK1 is observed in patients with intestinal infarction. miR146a lowers IRAK1 levels in human intestinal samples.

Experiments are well presented and the data shown support the conclusions reached by the authors. However at the level of originality and novelty the data presented do not advance what is currently known from previous literature. The TLR4/MyD88 is a pathway well described in intestinal I/R injury (Moses et al., 2009) and miR146a has recently been shown to mediate protective immune tolerance in the intestine via IRAK1 modulation (Chassin et al., 2010).

The report would be interesting if further work at the mechanistic level could be worked out. For example at the biochemical level there is no indication or validation of potential pathways operating downstream of IRAK-1 in intestinal injury nor is there an indication of whether, in vivo, the pathology induced is dependent on IEC-apoptosis due to IRAK-1 function in epithelial cells.

1st Revision - authors' response 02 July 2012

Editor's points

In particular, reviewers #1 and #2 highlight that the investigation of the therapeutic aspects of the study should be extended and make specific suggestions for that. Importantly, Reviewer #1 highlights that it is crucial to use hypoxic chambers instead of mineral oil to mimic hypoxia in vitro. Referee #3 feels that the mechanistic insight of the study is rather limited and suggest alternative paths that could be considered to strengthen the paper.

We have extended the study adding significant new experimental results following the suggestions made by reviewer 1 and 2. All *in vitro* results have been repeated in hypoxic chambers with very similar results as shown in new figure 1, 2 and 4A-C (see also response to major point 1, reviewer 1). Additional experiments now provide a more in depth mechanistic insight in the pathways involved in the mucosal damage observed in our *in vivo* I/R model as a consequence of enhanced Irak1 protein expression. New results indicate that Irak1-dependent activation of the Jnk signaling pathway leads to Bax phosphorylation which in turn mediates mitochondrial Aif release and nuclear translocation and caspase-independent apoptotic epithelial cell damage (new Fig. 3H-I, new Fig. 4J and new Fig. S3D; see also response to point 3, reviewer 2 and point 1, reviewer 3). MicroRNA-146a or DIM administration controls epithelial Irak1 expression and is able to prevent Jnk stimulation, Bax phosphorylation and Aif nuclear translocation consistent with our previous finding of its protective effect on mucosal tissue damage. Together with the identification of the molecular

© European Molecular Biology Organization

mechanism of hypoxia-induced Irak1 accumulation, namely an altered Irak1 ubiquitination pattern and SUMOylation, our results now provide a comprehensive description of the molecular process of post-ischemic innate immune hyper-responsiveness. Finally, the effect of DMOG administration on epithelial innate immune responsiveness and I/R injury has been evaluated. See specific comments below.

Reviewer 1 comments

This is an interesting manuscript which addresses a clinically important question. However some key issues need to be resolved. Firstly, the authors have previously demonstrated that IRAK-1 suppression is protective in bacteria-induced damage in the neonatal gut and that miR-146a targets IRAK-1 to provide protection. (Chassin et al 2010; Lotz et al. 2006). Therefore, some further in vivo experimentation is required to provide the necessary novelty to justify publication in EMBO Molecular Medicine. Secondly, from a technical point of view, I do not accept the use of mineral oil overlay as a mimic of hypoxia. In my opinion, these experiments should be done using a controlled environment chamber as is the current state-of-the-art in the field. Any in vitro work demonstrating the effects of hypoxia should use hypoxia chambers (and not oil immersion). Thirdly, the authors show that the hypoxia mimetic DMOG also increases IRAK1 expression in vitro. This would predict that DMOG would exacerbate IR injury whereas DMOG has previously been shown to be protective in intestinal IR injury (Hart et al J. Immunol 2011). Therefore the authors should test the effects of hydroxylase inhibition with DMOG in their model.

In this manuscript, Chassin et al. present data supporting a model which proposes that ischemia-reperfusion-induced IRAK-1 posttranscriptional upregulation leads to TLR-dependent immune hyper-reactivity associated with reperfusion injury in intestinal epithelial cells. The authors also suggest that delivery of miRNA 146a may represent a therapeutic intervention strategy in IRI. Understanding the immunologic basis and developing new therapeutics for reperfusion injury is an interesting and important area of research. The current manuscript contains some interesting data (especially the in vivo studies). However some significant experimental and methodological issues exist which need to be addressed.

<u>Major points:</u>
Point 1. *The use of mineral oil to elicit hypoxia in cells in culture is not, in my opinion, a valid technique. It is not possible to ascribe the effects of mineral oil to hypoxia alone and there is no appropriate negative control to account for the physical effects of the oil. In this study, anaerobic chambers were also used and some of this data is shown in the supplementary information, however it is not clearly detailed which experiments used anaerobic chambers and which experiments used mineral oil. In my opinion, the primary data reported should only be those generated using hypoxia/anaerobic chambers with an appropriately controlled environment and defined oxygen levels. Negative control cells should be cultured in an incubator with the same conditions (e.g. temperature, CO2) and a lower oxygen level.*

We do agree with the reviewer and have repeated all *in vitro* experiments using hypoxic chambers as recommended. All primary *in vitro* data shown in the revised version of the manuscript (i.e. all results shown in **new Fig. 1,2 and 4A-C**) have been generated using hypoxic chambers; the previously included data obtained using oil overlay have been removed or included in the supplemental information section. The figures 1, 2 and 4A-C in addition to the corresponding legends and manuscript text have been modified accordingly.

Point 2. *In figure S2B, the authors need to show that the knockdown of HIF-1 and HIF-2 was successful.*

As requested, we have included immunoblot experiments showing the downregulation of Hif-1 and Hif-2 following siRNA treatment (**new Fig S2B**).

Point 3. *The authors demonstrate that the hydroxylase inhibitor DMOG also upregulates IRAK-1 (curiously, this appears to be independent of HIF). As such, DMOG would be expected to exacerbate the effects of IR injury. Previous reports indicate that this is not the case and in fact that*

DMOG is protective (Hart et al., J. Immunol 2011). This begs the question, what is the impact of hydroxylase inhibition (e.g. DMOG administration) on IR injury in this model?

The hydroxylase inhibitor DMOG was used in our work to confirm the involvement of hydroxylase activity in Irak1 degradation. Enhanced Irak1 protein expression in DMOG treated cells (A) is in accordance with our results that suggest an involvement of SENP1 and SUMOylation in increased Irak1 protein expression during hypoxia.

Hart et *al*. report that DMOG upregulates expression of the ecto-5'nucleotidase (CD73) and the A2B adenosine receptor A2BAR and demonstrate that the protective effect of DMOG is abolished in the absence of either CD73 or A2BAR using *Cd73* and *A2bar* deficient mice, respectively. As suggested by reviewer 1, we have employed DMOG in *in vivo* experiments in our I/R model to evaluate its possible effect on I/R-mediated tissue injury in our model. DMOG has been injected in the lumen of the intestine (5 mg in 200µl total) prior to I/R treatment. As shown below, significant I/R-induced chemokine expression (B), epithelial barrier impairment (C), enhanced oxygen radical production (D), and tissue damage (illustrated by H&E and TUNEL staining in E and F) was also observed in the presence of DMOG. Yet, the protective effect of DMOG was reported to act via upregulation of CD73 and the adenosine receptor A2BAR, a mechanism independent of IRAK1-mediated signaling. Since the mechanism of CD73 and A2BAR–mediated protection was not investigated in our model, we prefered not to include these results in the revised version of the manuscript. A discussion of this point has been added to the discussion section of the revised manuscript (page 11, 2nd paragraph and 12, 1st paragraph).

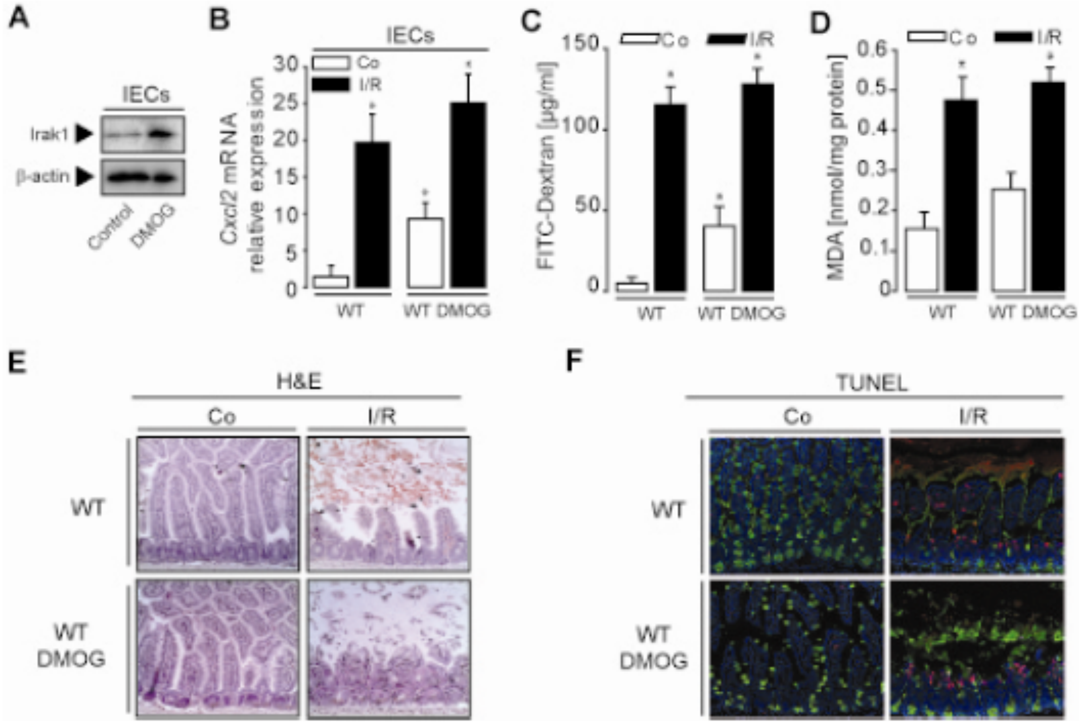

Point 4. *Are the effects of DIM specific for miR-146a in this model? Are other miRNAs not induced? The authors need to demonstrate this by examining the effects of DIM on other miRNAs.*

As suggested, we have analyzed the effect of DIM on additional microRNAs such as Let7a, miR-21, miR-155 and miR-29a known to be involved in the regulation of inflammation or I/R injury. Our results indicate that no other significant difference has been found in miR expression after DIM treatment in our model. In that case, the effect of DIM is restricted to the Irak1 regulating miR-146a and this finding has been added to the manuscript (**new Fig. S4B-E**).

<u>Minor Points</u>

Point 1. *Abstract line 1 typo- "damage" (not damages).*

The typo has been corrected.

© European Molecular Biology Organization

Point 2. *The labeling of Fig S1C and S1D do not match between the figure and the text.*

The labeling of Figure S1C and S1D has been corrected to match the reference in the text (page 5). We apologize for the mistake.

Point 3. In figure 2, was hypoxia induced using mineral oil or a hypoxia/anaerobic chamber? The experiments described here should be using the chamber system.

As suggested by the reviewer and described in the response to the major point 1 of reviewer 1, all experiments previously performed using the oil overlay model have now been repeated using the hypoxic chamber system. The results confirm our previous findings and are demonstrated in the new **Fig.1, 2 and 4A-C** (see also comment to major point 1, 1st reviewer).

Reviewer 2 comments

Of potential interest. However, the manuscript need further experiments to support claims of therapeutic effects of micrRNA146a or DIM before accepting publication.
In this manuscript the authors initially seek to establish and characterize the involvement of IRAK1 as part of the TLR response, in tissue damage following I/R insult in the intestinal cells, mouse intestines and human intestinal explants. This part of the manuscript is very interesting, convincing and thorough, and the manuscript is very well written. The authors subsequently focus on therapeutic strategies using (miR)-146a to address I/R injury via stabilization of IRAK1, which is of potential interest. However the observation is not novel as the authors themselves and others have already shown IRAK1 regulation by (miR)- 146a. As the scope of the manuscript is intended to be therapeutic, the following issues would be needed in order to strengthen the therapeutic effect of (miR)-146a and DIM on I/R injury.

Major points:
Point 1. *The major concern of this manuscript, given the title presented - "the therapeutic effect of (miR)- 146a or DIM on I/R injury", is that it is not adequately substantiated and in part inconclusive. More in vivo experiments using mice, assessing dose dependant effect of DIM or (miR)- 146a, oral administration (gavage) of DIM post I/R injury i,e evaluating its therapeutic effect (post injury) needs to be analyzed.*

DIM acts via miR-146a to prevent enhanced Irak1 protein expression. Thus DIM administration only after completion of hypoxia (and thus when Irak1 protein levels are already strongly enhanced) is not expected to provide protection from I/R injury. We agree with the reviewer that this suggests a possible prophylactic rather than therapeutic use of DIM. Since the data provided may not justify the therapeutic option we have modified the title of our manuscript (page 1, line 3 and 4).

Additionally, we have added new *in vivo* experimental data that strengthen the conclusion of a significant effect of DIM on Irak1 mediated hyper-responsiveness and signaling-mediated epithelial cell damage following ischemia/reperfusion *in vivo* and allow a more complete understanding of the DIM and microRNA-146a-mediated effect as recommended (**new Fig. 3H and I, Fig, 4J**).

Point 2. *In classical I/R injury, reactive oxygen species are thought to be the major cause of tissue damage, furthermore IRAK1 has also been shown to induce ROS generation. It would be valuable in this context to assess the levels of ROS like lipid peroxidation or reduced glutathione (GSH) or hydroxyl radicals, pre and post DIM treatment in mice.*

As suggested we have evaluated the lipid peroxidation as a consequence of inappropriate Irak1-mediated innate immune signaling and oxidative injury (**new Fig. 4G**) in the absence or presence of DIM or microRNA-146a administration. As proposed by the reviewer, our results indeed suggest enhanced oxygen radical formation as a consequence of increased Irak1 mediated signaling. Also DIM or microRNA-146a administration reduce the degree of lipid oxidation in accordance with our previous findings.

Point 3. *The Tunel assay immunostainings especially in figures 3F and 4F are less convincing enough and needs to be replaced by better quality images with higher magnification and resolution. Moreover, the authors appear not to consider the possibility of necrosis that is quite likely to be taking place in parallel to apoptosis during I/R. It is equally possible that there could be more of*

© European Molecular Biology Organization

necrosis than apoptosis under these circumstances. Since Tunel assay fails to discriminate between the two processes of cell death, it may be further substantiated with assays specific for apoptosis or necrosis and this issue should be adequately discussed.

As recommended, the TUNEL images in Fig. 3 and 4 have been improved/replaced to provide better quality as requested. Also, we agree with the reviewer's comment that the TUNEL assay does not discriminate between apoptosis and necrotic cell death and have performed additional experiments to better characterize the I/R injury. Specifically, we have have performed immunostaining for active caspase 3 (**new Fig. S3D**), immunoblotting in total cell lysate of isolated IECs from wildtype and Irak1 deficient mice following I/R for Bax, phospho Bax, Jnk and phospho Jnk (**new Fig. 3H, and Fig. 4J**), immunostaining for Jnk phospho Jnk, Bax, phospho Bax in total cell lysate of isolated IECs from mice following I/R without or with DIM or miR-146a mimic administration (**new Fig. 4J**) as well as immunostaining for Aif in nuclear and cellular extract of isolated IECs from wildtype or Irak1 deficient mice after I/R without or with miR-146a or DIM administration (**new Fig. 3I and Fig. 4J**). The new results suggest that hypoxia-mediated Irak1 upregulation leads to stimulation of the Jnk signaling pathway and Bax phosphorylation, which induces mitochondrial release and nuclear translocation of Aif with subsequent caspase 3-independent apoptosis of the intestinal epithelium.

Point 4. *The sentence in results section "A significant, time-dependent increase in IRAK1 was observed during the course of oxygen deprivation using two different models (Fig. 1A and S1C)." is misleading as in both the figures the same model "oil overlay" has been used. Moreover, S1C shows no time dependence at all.*

This point was due to a mislabeling of the figures in the original version of the results section. The figure reference in the original version should have read "Fig. 1A and S1D" since these two immunoblot analyses indicate increased Irak1 protein levels after hypoxia (Fig. 1A and Fig. S1D). Fig. S1D also includes time dependence as mentioned in the text. We apologize for the mistake and have carefully revised the modified version of the revised manuscript.

Point 5. *In the results section, in line "Induction of hypoxia was confirmed by enhanced expression of the hypoxia-inducible factor (HIF)-α; analyzed by immuno-blotting and immuno-staining (Fig. 1A and S1D)" either of the mentioned figures does not show any immuno-staining. These discrepancies between results and figures are confusing and needs to be corrected.*

The reviewer is correct. We apologize for the misunderstanding, which was due to a mislabeling of the panels in Fig. 1 and Fig.S1 of the original version of the manuscript. Enhanced expression of Hif1α in hypoxic epithelial cells is now shown by immunoblotting and immunstaining in the **new Fig. 1A and Fig. S1C**, respectively.

Point 6. *In the sentence "Interestingly, the increase in IRAK1 protein was prevented in the presence of the miR-146a inducing agent, DIM (Fig. S5B).", the mentioned figure S5B does not exist rather should be 5B. The authors need to properly revise the manuscript in order to correct this and other similar mistakes.*

Again, we apologize for the mislabeling, which has caused the misunderstanding. The effect of DIM on Irak1 protein expression in human IECs after hypoxia is shown in **Fig. 5B** in the revised manuscript.

Reviewer 3 comments

In this manuscript the authors show that: 1. Hypoxia in IECs in vitro leads to increased IRAK1 levels, increased MIP2 expression and sensitization to LPS; 2. Hypoxia in IECs in vitro leads to an alteration of the IRAK1 K48/K63 ubiquitination ratio and induces IRAK1 sumoylation; 3. IRAK1 and MIP2 are increased in I/R injury. IRAK1-/- mice show attenuated I/R injury pathology, decreased gut permeability and apoptosis; 4. Induction of miR146a reduces IRAK1 and MIP2 levels in-vitro and in-vivo and leads to attenuated I/R injury, decreased gut permeability and apoptosis; 5. Increased IRAK1 is observed in patients with intestinal infarction. miR146a lowers IRAK1 levels in human intestinal samples.

Experiments are well presented and the data shown support the conclusions reached by the authors. However at the level of originality and novelty the data presented do not advance what is currently known from previous literature. The TLR4/MyD88 is a pathway well described in intestinal I/R injury (Moses et al., 2009) and miR146a has recently been shown to mediate protective immune tolerance in the intestine via IRAK1 modulation (Chassin et al., 2010).

Major point

Point 1. *The report would be interesting if further work at the mechanistic level could be worked out. For example at the biochemical level there is no indication or validation of potential pathways operating downstream of IRAK-1 in intestinal injury nor is there an indication of whether, in vivo, the pathology induced is dependent on IEC-apoptosis due to IRAK-1 function in epithelial cells.*

As recommended (and also requested by reviewer 2, point 3) we have performed additional experiments to obtain deeper insight into the mechanisms involved in the downstream pathways involved in the observed I/R-induced epithelial cell damage. In particular, we have analyzed the cleavage of caspase 3 in I/R tissue (**new Fig. S3D**), stimulation of the Jnk signaling pathway and phosphorylation of the apoptosis promoting Bax in wildtype and Irak1 deficient mice after I/R (**new Fig. 3H**) and the nuclear translocation of the apoptotic mediator Aif (**new Fig. 3I**). These results also demonstrate the inhibitory effect of DIM or miR-146a administration on Jnk and Bax phosphorylation and Aif translocation (**new Fig. 4J**). The new results suggest that I/R injury in our model is associated with enhanced Jnk signalling, Bax-phosphorylation, Aif release and nuclear translocation leading to caspase 3-independent epithelial apoptosis (see also reviewer 2, point 3).

2nd Editorial Decision 25 July 2012

Thank you for the submission of your revised manuscript to EMBO Molecular Medicine. We have now received the enclosed report from the referee who was asked to re-assess it.

As you can see, this referee is still not fully satisfied with the data and is especially asking for the DMOG experiment in vivo to be repeated iv or ip in order to make the results comparable to previous published data using the same method.

We would welcome the submission of a revised version for further consideration, providing that all points are addressed, and depending on the nature of the revisions, this may be sent back to the referee for another round of review.

On a more editorial matter, we appreciate that you added the table of content to the Supplementary Information. However, we note that the quality of the current Supplementary figure images is a bit low. Please provide higher resolution versions, and check to make sure that text/line-art remains clear even when zooming in. You may find that saving the images as EPS or PDF will better preserve the text and line-art resolution. If this does not help, you may need to remake the figures in a quality vector graphics program like Illustrator or the free opensource, alternative Inkscape.

Please submit your revised manuscript within 3 months. I look forward to seeing a revised form of your manuscript as soon as possible.

Yours sincerely,

Editor
EMBO Molecular Medicine

***** Reviewer's comments *****

Referee #1 (Comments on Novelty/Model System):

The administration of DMOG in the experiment to examine the therapeutic implications of the current study is intra-luminal (data presented only in response to reviewer). I do not understand the

© European Molecular Biology Organization

rationale for this as previous studies have demonstrated the effectiveness of systemic administration of this dose. Putting such a high dose directly into the lumen may cause local injury and mask any protective effects of the drug. As the data presented in the manuscript contradicts a number of models showing DMOG to be protective in various models of intestinal inflammation, the experiment should be repeated using an IP dose.

Referee #1 (Other Remarks):

The authors have addressed some of my concerns, however, there are still some key issues remaining.

1) The authors have now used anaerobic / hypoxic chambers to repeat the "mineral oil immersion" experiments. As I stated in my original review, I believe the mineral oil immersion method to achieve hypoxia to be invalid and I think most researchers in the field of hypoxia would agree. In my opinion, this method should be removed from the supplemental data as it adds little in the way of useful information. The numerical data obtained in the new experiments using the hypoxia chambers are remarkably similar to the data generated by the oil immersion method in the first submission of the manuscript (e.g figure 2B, 2C, 2D, Fig 4A). Can the authors confirm that all in vitro data was obtained using the hypoxia chambers to achieve hypoxia. Furthermore, what was the method used to achieve hypoxia in the human biopsies in Figure 5B and 5C?

2) With regard to the chambers used to achieve hypoxia, it is vital that the authors describe the conditions used clearly. For example, what are the types of the chambers used? What atmospheric conditions are they using to achive hypoxia and contol normoxia (what is the percent atmospheric oxygen-what other gasses are present). This is important to assess whether this is an appropriate and valid experimental system.

3) In my initial review, I suggested that the authors use DMOG in vivo to examine the possible therapeutic implications of the study. The authors carried out these studies and for some reason used an intraluminal injection of 5 mg DMOG rather than a systemic (I.P. or I.V.) injection which have now been shown in a number of studies to be effective in the treatment of various inflammatory injuries to the intestine (e.g. Hart et al., J. Immunol 2011, Cummins et al. Gastroenterology 2008, Robinson et al. Gastroenterology 2008, Hindryckx et al. J Immunol, 2010, Hiroto et al. Gastroenterology 2010). The intraluminal dose used in this study may well have resulted in such high local concentrations as to cause tissue injury. This makes the current study difficult to compare to previous studies. I suggest repeating this experiment using IP administration of DMOG as per previous studies. In the absence of this experiment, the therapeutic relevance of the current study is limited.

2nd Revision - authors' response 21 September 2012

Editor's points

Regarding point 1 raised by the reviewer, we agree, that the it would be very time- consuming to repeat the experiments using human biopsies in a hypoxic chamber.
Since your results indicate that hypoxic chamber and oil overlay have similar effects, these experiments would not be a requirement for further consideration at EMBO Molecular Medicine. We would encourage you, however, to extend your Material and Methods section and clearly describe, which conditions were used in which experiment. This would also address the point 2 raised by the reviewer.
As recommended we have extended our Material and Methods section and now provide a detailed description of the precise conditions used for each individual experiment (page 14, 2nd paragraph and legends of each figure). In agreement with the editor we did not repeat the analyses with human biopsy samples using anaerobic chambers. The editor's argumentation is illustrated by the similar resulted obtained with both methods in vitro (e.g. Fig. 1 versus Suppl. Fig. 1).

© European Molecular Biology Organization

Regarding point 3 raised by the reviewer, we further considered the issue and feel that we can accept it when you discuss this point as appropriate in the manuscript
We included a new paragraph discussing our data using local DMOG administration in the context of the previously published results using i.v. or i.p. injection of DMOG in I/R and other mucosal inflammation models in the revised manuscript (page 11, last paragraph – page 12, first paragraph). Also, we added citations of the relevant literature indicated by the reviewer (except the listed publication "Hiroto et al., Gastroenterology, 2010" which we were unable to identify).

Finally, the statistical test used in each figure has been specified and the requested additional statistical information has been added to the legend text. Also, the resolution of the supplementary figure images has been increased as requested.

Reviewer 1 comments

General remark:
The administration of DMOG in the experiment to examine the therapeutic implications of the current study is intra-luminal (data presented only in response to reviewer). I do not understand the rationale for this as previous studies have demonstrated the effectiveness of systemic administration of this dose. Putting such a high dose directly into the lumen may cause local injury and mask any protective effects of the drug. As the data presented in the manuscript contradicts a number of models showing DMOG to be protective in various models of intestinal inflammation, the experiment should be repeated using an IP dose.

Please see response to specific point 3.

Specific points raised:
1. The authors have now used anaerobic / hypoxic chambers to repeat the "mineral oil immersion" experiments. As I stated in my original review, I believe the mineral oil immersion method to achieve hypoxia to be invalid and I think most researchers in the field of hypoxia would agree. In my opinion, this method should be removed from the supplemental data as it adds little in the way of useful information. The numerical data obtained in the new experiments using the hypoxia chambers are remarkably similar to the data generated by the oil immersion method in the first submission of the manuscript (e.g figure 2B, 2C, 2D, Fig 4A). Can the authors confirm that all in vitro data was obtained using the hypoxia chambers to achieve hypoxia. Furthermore, what was the method used to achieve hypoxia in the human biopsies in Figure 5B and 5C?

As confirmed by the reviewer we had repeated all *in vitro/ex vivo* mouse experiments using hypoxic chambers for the first revision of the manuscript. The re-revised manuscript now specifies the technical details for all presented experiments individually as requested. The suggestion to repeat the analyses of human biopsy samples shown in Fig. 5 has been discussed with the editor. The editor agreed to show the current results given (i) the high similarity of the results obtained with both methods using murine cells (e.g. Fig. 1 versus Suppl. Fig. S1) and (ii) the difficulties to obtain a renewed ethical statement and additional human biopsy material.

2. *With regard to the chambers used to achieve hypoxia, it is vital that the authors describe the conditions used clearly. For example, what are the types of the chambers used? What atmospheric conditions are they using to achive hypoxia and contol normoxia (what is the percent atmospheric oxygen-what other gasses are present). This is important to assess whether this is an appropriate and valid experimental system.*

The conditions using anaerobic chambers or ambient atmosphere are now described in great detail in the Material Methods section (page 14, 2nd paragraph and legends to each figure). We repeated the control measurements of our technical equipment again and confirmed the O_2 and CO_2 concentrations indicated in the Material and Methods section.

3. In my initial review, I suggested that the authors use DMOG in vivo to examine the possible therapeutic implications of the study. The authors carried out these studies and for some reason used an intraluminal injection of 5 mg DMOG rather than a systemic (I.P. or I.V.) injection which have now been shown in a number of studies to be effective in the treatment of various inflammatory

injuries to the intestine (e.g. Hart et al., J. Immunol 2011, Cummins et al. Gastroenterology 2008, Robinson et al. Gastroenterology 2008, Hindryckx et al. J Immunol, 2010, Hiroto et al. Gastroenterology 2010). The intraluminal dose used in this study may well have resulted in such high local concentrations as to cause tissue injury. This makes the current study difficult to compare to previous studies. I suggest repeating this experiment using IP administration of DMOG as per previous studies. In the absence of this experiment, the therapeutic relevance of the current study is limited.

This issue has been discussed with the editor who agreed that we do not have to perform the requested i.v./i.p. DMOG experiment since the identical experiment has already been performed and published (Hart et al., J. Immunol., 2011). Importantly, we used the hydroxylase inhibitor DMOG in our original study only under normoxic conditions to demonstrate the involvement of hydroylases to maintain IRAK1 protein levels in the presence of oxygen. Also, we would like to stress that we did not observe any significant local tissue damage attributed to DMOG administration *in vivo* as illustrated by the images provided with the response letter of the first revision. Finally, since our main interest is the gut epithelium, we favored the local administration in order to avoid an influence on systemic responses. Local DMOG administration appears feasible and might even potentially be associated with less toxic effects. This issue is now discussed in the manuscript (page 11, last paragraph – page 12, first paragraph).

3rd Editorial Decision 25 September 2012

(All requested changes have been made)

© European Molecular Biology Organization
