# Supplementary material for: MicroRNA-146a-mediated downregulation of IRAK1 protects mouse and human small intestine against ischemia/reperfusion injury
Source: EMBO Mol Med. 2012 Nov 9;4(12):1308–19. doi: 10.1002/emmm.201201298 (PMC3531605; doi:10.1002/emmm.201201298)
Supplement: Supplementary file 2 [file emmm0004-1308-SD2.pdf]

Supplementary Information

Table of Contents

Supplementary Materials and Methods …………………………………..…………………………………. 2

Fig. S1. Enhanced LPS susceptibility precedes hypoxia-induced epithelial damage ………………………. 3

Fig. S2. Irak1 accumulation is not regulated at a transcriptional level and is independent of Hif-1/2……...4

Fig. S3. Irak1 increases in IECs after I/R injury…………………………………...………………………. 5

Fig. S4. Expression of microRNAs in IECs after hypoxia………………………...………………………. 6

Fig. S5. DIM decreases hypoxia-induced innate immune hyper-responsiveness of human intestine……… 7

Supplementary Materials and Methods

Experimental intestinal I/R and IEC isolation. For ischemia/reperfusion (I/R), mice were anesthetized, the abdominal cavity was shaved, opened and a 4-cm long segment of the small intestine was ligated with a 4-0 silk suture. The corresponding mesenteric artery was clamped to interrupt the blood supply using thin surgical vascular clamps suitable for mice. The surgical procedure was performed on a heated-table thermostated at 38°C appropriate for small animals for ~5 min. After 30 min, the clamp was removed, the abdominal skin sutured, and the tissue was subjected to reperfusion for 60 min. The animals were then sacrificed, and the ischemic/reperfused intestinal tissue, as well as an unaffected proximal segment of the small intestine (control tissue), was collected for histological analysis or IEC isolation as previously described (22). For *ex-vivo* stimulation experiments, I/R was performed and small-intestinal tissue was removed, extensively rinsed and incubated in the absence or presence of 100 ng/mL LPS at 37°C for 2 h. Subsequently, IECs were isolated as previously described (22). For luminal exposure, 200 µl of 100 ng/mL LPS or medium control were injected into the intestinal lumen of a segment of healthy control intestinal tissue, or of a segment of intestinal tissue that had been subjected to I/R. After 2 h, the animals were sacrificed and IECs were isolated. DIM (200 µl of a 25 µM solution) or miR-146a miRIDIAN mimic (1 nM) was administered intra-luminally prior to ischemia.

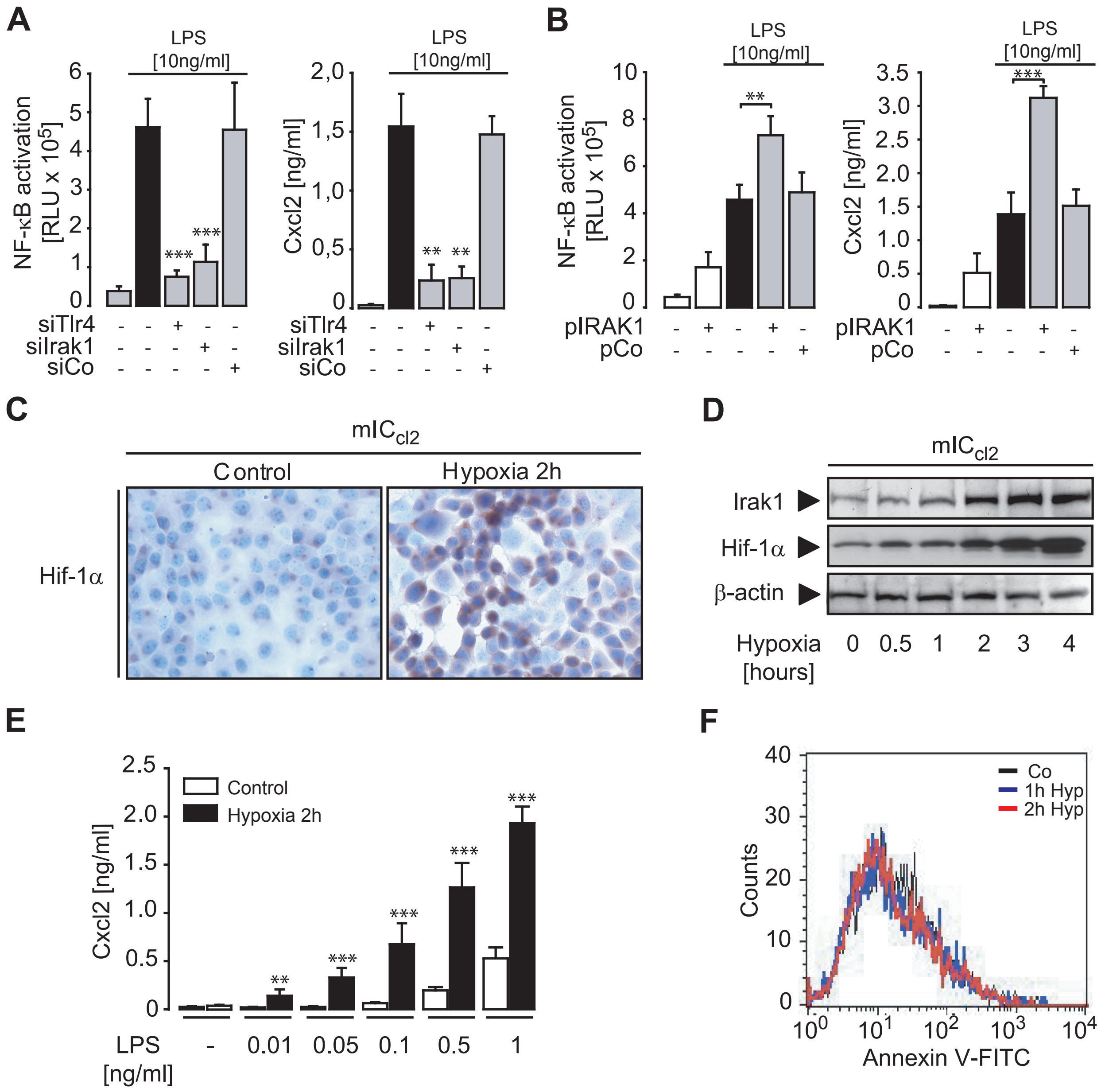

Fig. S1. Enhanced LPS susceptibility precedes hypoxia-induced epithelial damage. (A-B) m-IC$_{cl2}$ cells were treated with siRNA against *Tlr4* or *Irak1* prior to stimulation with 10 ng/mL LPS (A), or transiently transfected with an *Irak1* encoding plasmid (B), and activation of a NF-κB luciferase reporter (A: siTlr4+LPS 7526 ± 1588 and siIrak1+LPS 11326 ± 4475 *vs* LPS 46151 ± 7335, p = 0.0008; B: pIRAK1+LPS 75435 ± 13985 *vs* LPS 44096 ± 6134, p = 0.02) as well as secretion of the chemokine Cxcl2 were quantified (A: siTlr4+LPS 216 ± 115 and siIrak1+LPS 248 ± 95 *vs* LPS 1515 ± 390, p = 0.005; B: pIRAK1+LPS 3122 ± 73 *vs* LPS 1386 ± 325, p = 0.0008). n = 3 for each data point. **(C)** mIC$_{cl2}$ cells were subjected to hypoxia by overlaying with mineral oil for 2h and Hif-1α was immunostained (Magnification ×400). **(D-E)** mIC$_{cl2}$ cells were subjected to hypoxia by overlaying with mineral oil for the indicated time and (D) Irak1 and Hif-1α levels were checked by immunoblot, or (E) cells were incubated with LPS for 6h under normoxic conditions (n = 5 for each data point; 0.01: 140 ± 65 *vs* 20 ± 6, p = 0.01; 0.05: 329 ± 99 *vs* 24 ± 10, p = 0.0008; 0.1: 675 ± 217 *vs* 63 ± 10, p = 0.001; 0.5: 1265 ± 253 *vs* 196 ± 34, p = 0.0001; 1: 1932 ± 171 *vs* 528 ± 113, p = 0.000009). **(F)** The cellular viability after 1 and 2 h hypoxia was assessed by annexin V staining and flow cytometric analysis. ***Student's t-test p <0.001, ** p <0.05 between groups. Values are means ± SEM from 3-5 separated experiments.

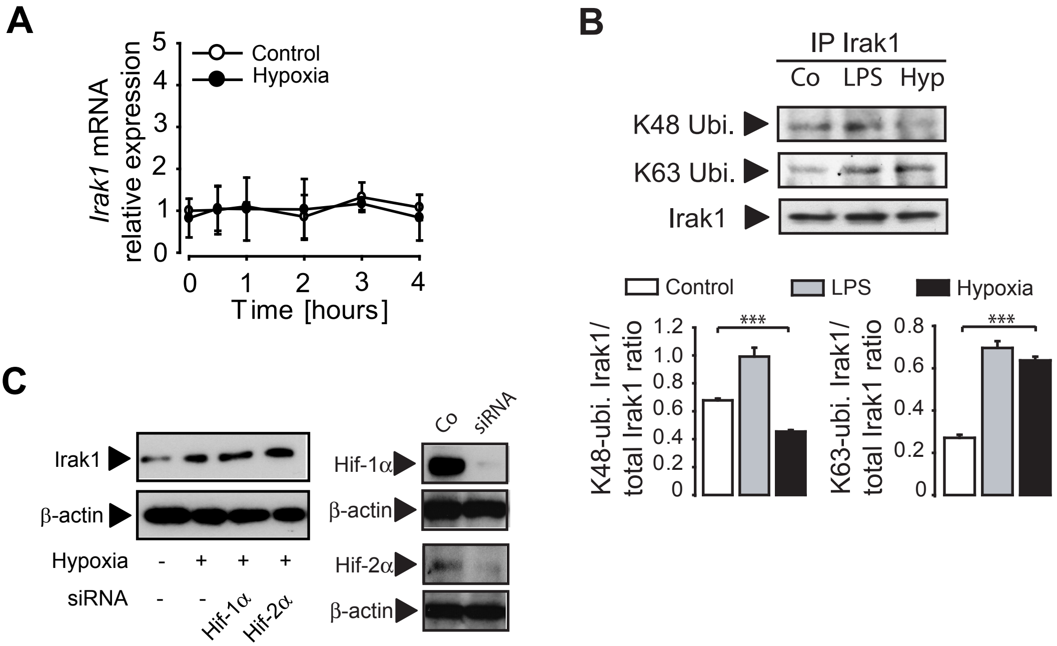

Fig. S2. Irak1 protein accumulation is not regulated at a transcriptional level and is independent of Hif-1/2. (A) mIC$_{cl2}$ cells were subjected to hypoxia by oil overlay for various times and *Irak1* mRNA expression was quantified by real-time PCR. Values are means ± SEM from 4 separated experiments. **(B)** mIC$_{cl2}$ cells were transfected with an ubiquitin-encoding plasmid and subjected to hypoxia (2h oil overlay) or treated with 10 ng/mL LPS for 2 h in the presence of the proteasome inhibitor MG132 (100 nM). Irak1 was immunoprecipitated, and K48 or K63 ubiquitination was individually detected by immunoblot and quantified from four independent experiments (n = 3 for each data point; K48-ubi: 0.4543 ± 0.0112 vs 0.6775 ± 0.0134, p = 0.00002; K63-ubi: 0.6375 ± 0.0172 vs 0.2711 ± 0.0141, p = 0.000009). ***Student's t-test p <0.001 between groups. Values are means ± SEM from 3 separated experiments. **(C)** mIC$_{cl2}$ cells were treated with siRNA against *Hif-1*α or *Hif-2*α prior to hypoxia for 2h, then Irak1 level was detected by immunoblot. The silencing of *Hif-1*α and *Hif-2*α was controlled by immunoblot.

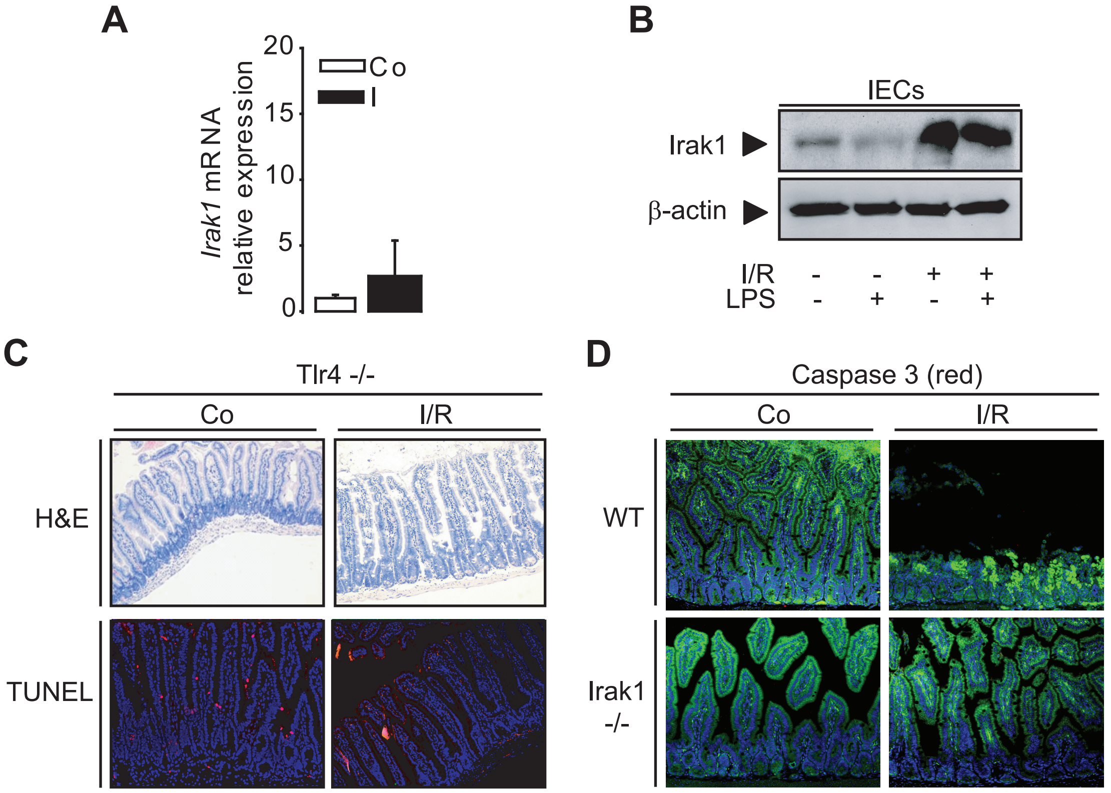

Fig. S3. Irak1 increases in IECs after I/R injury. Small intestinal tissue of wild-type mice was subjected to I/R as described. Ischemic intestinal tissue and unaffected control tissue was removed and incubated for 2 h in the presence of 100 ng/mL LPS at 37°C. Subsequently, IECs were isolated, **(A)** *Irak1* mRNA expression was quantified by real-time PCR, and **(B)** Irak1 protein expression was determined by immunoblotting. Values are means ± SEM from 4 separated experiments. **(C)** Small intestinal tissue of *Tlr4*-/- mice subjected to I/R were stained with H&E or TUNEL. **(D)** Caspase 3 staining (red) was negative in small intestinal tissue of wild-type mice subjected to I/R. Magnification ×100.

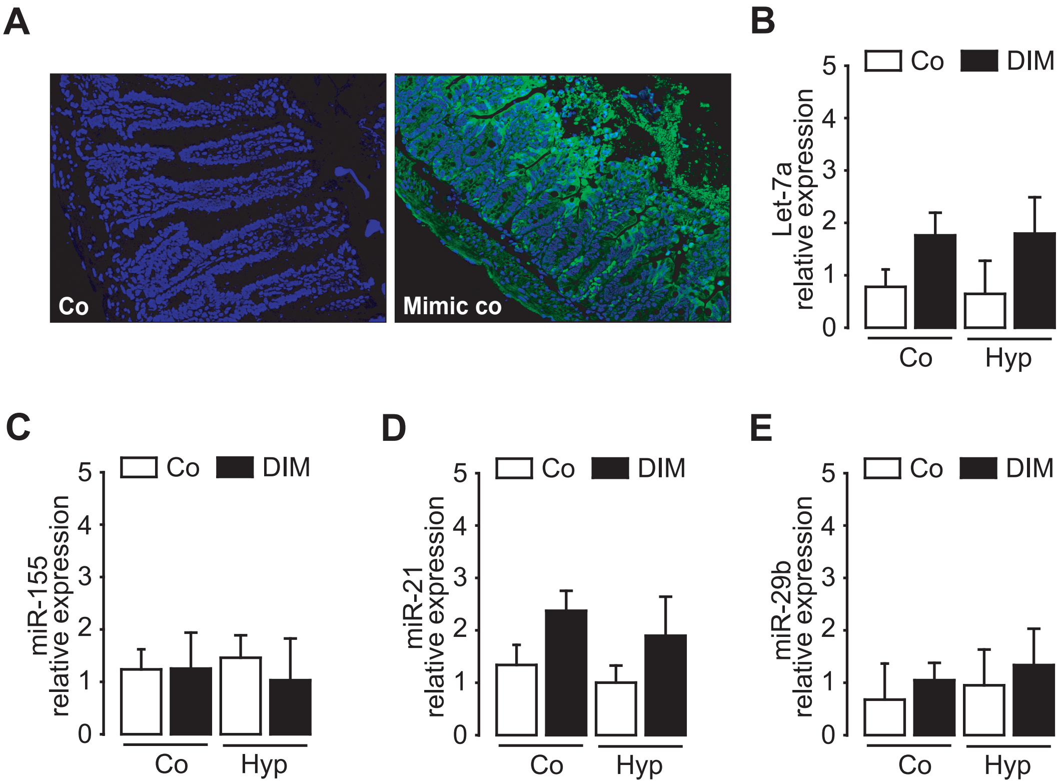

Fig. S4. Expression of microRNAs in IECs after hypoxia. (A) Uptake of miR mimic by intestinal tissue after intraluminal injection. Cy3-labelled miR mimic control (1 nmol, Mimic co, right panel) or solvent control (co, left panel) was injected intraluminally into the small intestine, and the tissue was then subjected to I/R. Uptake of miR mimic co (green, false color) was visualized by immunofluorescence. Counterstaining with Dapi (blue). Magnification ×200. **(B-E)** mIC$_{cl2}$ cells were subjected to hypoxia in hypoxic chambers for 2h (Hyp) or not (Co) and incubated with DIM (black bars) or not (white bars), and the level of let-7a (B), miR-155 (C), miR-21 (D) and miR-29b (E) were determined by quantitative PCR. Values are means ± SEM from 5 separated experiments.

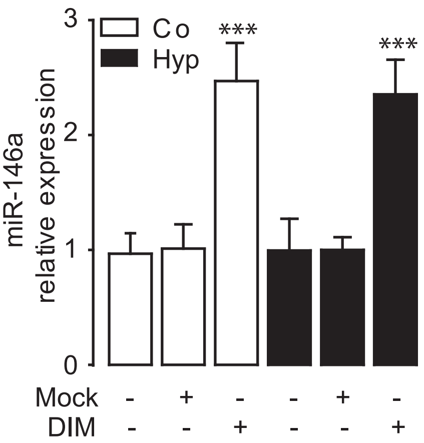

Fig. S5. DIM treatment decreases hypoxia-induced innate immune hyper-responsiveness of human intestine. Human biopsies (n=5) were left untreated (Co) or subjected to hypoxia (Hyp, oil overlay) in the absence or presence of 25 µM DIM or of solvent control (Mock) and miR-146a expression was determined by real-time RT-PCR. DIM (2.4688 ± 0.3122) vs control (0.9658 ± 0.1791), p = 0.0001. DIM + hypoxia (2.3530 ± 0.3011) vs hypoxia (0.9990 ± 0.1105), p = 0.0005. ***Student's t-test p <0.001, values are means ± SEM from 5 individual experiments.
